# A Taxonomic Revision of the *Dichotomius depressicollis* (Harold, 1867) Species Group (Coleoptera: Scarabaeidae: Scarabaeinae), with Description of Five New Species

**DOI:** 10.1007/s13744-026-01369-8

**Published:** 2026-04-08

**Authors:** JA Arias-Buriticá, FZ Vaz-de-Mello

**Affiliations:** 1 Programa de Pós-Graduação em Ecologia e Conservação da Biodiversidade, Universidade Federal de Mato Grosso, Cuiabá, Mato Grosso Brazil; 2 Depto de Biologia e Zoologia, Instituto de Biociências, Univ Federal de Mato Grosso, Instituto Nacional de Coleoptera (INCol), Cuiabá, Mato Grosso Brazil

**Keywords:** Taxonomy, *Dichotomius* (*Dichotomius*), New species, South America

## Abstract

Recently, the taxonomy of *Dichotomius* Hope, 1838 (Coleoptera: Scarabaeidae: Scarabaeinae) was updated, a new species group division was proposed, and the *Dichotomius depressicollis* (Harold, 1867) species group was created. Based on the external morphology and the male genitalia of all type material and 1443 specimens deposited in eight entomological collections, the taxonomic revision of this group is carried out. The group contains eight species, five of which are new: *Dichotomius audinoae* **sp. nov.** (Brazil), *Dichotomius barberoi* **sp. nov.** (Brazil), *Dichotomius davidedmondsi* **sp. nov.** (Bolivia), *Dichotomius depressicollis* (Harold, 1867) (Argentina and Brazil), *Dichotomius inca* **sp. nov.** (Peru), *Dichotomius melzeri* (Luederwaldt, 1922) (Bolivia, Brazil, and Peru), *Dichotomius salomaoi* **sp. nov.** (Brazil), and *Dichotomius zikani* (Luederwaldt, 1922) (Bolivia and Brazil). Lectotypes were designated for *Pinotus depressicollis* Harold, 1867, *Pinotus melzeri* Luederwaldt, 1922, and *Pinotus zikani* Luederwaldt, 1922. A complete description of the *D. depressicollis* species group and a species identification key are presented. For each species, the following information is provided: taxonomic history, description or redescription, list of material examined, photographs of external morphology, male genitalia and its endophallites, and distribution map.

## Introduction

*Dichotomius* Hope, 1838 is one of the most diverse genera of dung beetles (Coleoptera: Scarabaeidae: Scarabaeinae) with more than 200 described species distributed throughout the New World (Nunes and Vaz-de-Mello 2019; Cupello et al. 2023). Luederwaldt (1929) proposed a new approach to the infrageneric classification of the genus by dividing it into subgenera and sections (now called species groups), based on characters of external morphology. In recent years, different authors have highlighted some problems in Luederwaldt’s proposal; by including new specimens and new characters (mainly of the male genitalia), the groups (sections) proposed by this author present limits that are difficult to maintain, which is why the taxonomy of the group has varied in recent years (Arias-Buriticá and Vaz-de-Mello 2019, 2023, 2024a, 2024b; Moura et al. 2025a, 2025b; Nunes and Vaz-de-Mello 2019; Rossini and Vaz-de-Mello 2020; Valois et al. 2017, 2023, 2022).

Recently, the taxonomy of *Dichotomius* Hope, 1838 (Coleoptera: Scarabaeidae: Scarabaeinae) was updated, a new species group division was proposed and the genus was divided into five subgenera: *Dichotomius *sensu stricto, *Dichotomius* (*Cephagonus*) Luederwaldt 1929, *Dichotomius* (*Homocanthonides*) Luederwaldt 1929, *Dichotomius* (*Luederwaldtius*) Arias-Buriticá and Vaz-de-Mello 2025, and *Dichotomius* (*Selenocopris*) Burmeister, 1846. In the subgenus *D*. sensu stricto twelve species groups and two *incertae sedis* species were proposed (Arias-Buriticá and Vaz-de-Mello 2025). Recently, Moura et al. (2025b) described a third species assigned to the *incertae sedis* category for the subgenus. One of these groups is the *Dichotomius depressicollis* (Harold 1867) species group with three described species: *D. depressicollis*, *Dichotomius melzeri* (Luederwaldt 1922), and *Dichotomius zikani* (Luederwaldt 1922).

In this paper, we present a taxonomic revision of the *D*. *depressicollis* species group *sensu* Arias-Buriticá & Vaz-de-Mello (2025), based on external and male genital morphology. This revision includes all the type material and specimens of eight entomological collections. The lectotypes for *Pinotus depressicollis* Harold 1867, *Pinotus melzeri* Luederwaldt 1922, and *Pinotus zikani* Luederwaldt 1922 are designated. A species identification key is presented, and for each species, the complete information and photographs are included. It also includes the description of five new species from Bolivia, Brazil, and Peru.

## Materials and Methods

This study was based on the examination of 1443 specimens deposited in the following entomological collections (names of the curators and collaborators of each collection who assisted the authors during their visits are in parentheses). Collections with an asterisk next to their abbreviation are those where only the type material was reviewed:

**CALT-ECC**: Colección Escarabajos Coprófagos de Colombia, Bogotá, Colombia (Alejandro Lopera Toro and Arturo González).
**CEMT**: Coleção Entomológica de Mato Grosso Eurides Furtado, Cuiabá, Mato Grosso, Brazil (Fernando Vaz–de–Mello).
FIOC*: Coleção Entomológica do Instituto Oswaldo Cruz, Fundação Oswaldo Cruz, Rio de Janeiro, Brazil (Márcio Felix).
**MHNNKM**: Museo de Historia Natural Noel Kempff Mercado, Colección entomológica, Santa Cruz de la Sierra, Bolivia (Julieta Ledezma).
MNHN*: Muséum National d’Histoire Naturelle, Paris, France (Olivier Montreuil and Antoine Mantilleri).
**MUSM**: Museo de Historia Natural Universidad Mayor de San Marcos, Lima, Peru (Mabel Alvarado and Luis Figueroa).
MZSP*: Museu de Zoologia da Universidade de São Paulo, São Paulo, Brazil (Sonia Casari and Carlos Campaner).
**NMNH**: National Museum of Natural History, Washington, USA (Terry Erwin, material provided by Trond Larsen).

Species identification was performed using identification keys (Luederwaldt 1929) and original descriptions, as well as by examining all type specimens (Harold 1867; Luederwaldt 1922). For species for which there is no designated holotype in the original description, lectotypes have been designated in accordance with Article 74.7.3 and Declaration of the ICZN 44 (1999).

The dissection and preparation of the male genitalia (aedeagus) followed the methodology of Zunino (1978). Photographs of the structures of the external and internal morphology of males and females were taken using a Leica motorized stereomicroscope system model M205C with image capture system MC190 HD. Body measurements and scale bars were traced using Leica software. Terminology for external morphology follows Vaz-de-Mello et al. (2011), Lawrence et al. (2010) (mainly for the pterothorax and abdomen), and Nunes and Vaz-de-Mello (2019). Terminology of tegmen follows Nunes and Vaz-de-Mello (2019). The nomenclature of the male genitalia mainly follows the proposal of Tarasov and Solodovnikov (2011), with the following exceptions: the term “endophallite” proposed by Génier (2019) replacing “sclerite.” The term “medial endophallite” replaces “lamella copulatrix” (Medina et al. 2013, modified by Cupello et al. (2022)).

For each species, the taxonomic treatment developed shows the following information: location of the type specimens, nomenclatural history, material examined, diagnosis, description or redescription of the male and female, comments, and geographical distribution. Distribution maps were made in Quantum GIS (QGis version 3.16.8-Hannover). In the material examined, information was extracted *ipsis litteris* from the labels. The characteristics of the type labels are described before the label’s information in braces brackets (e.g., {quadrangular, red label}) and supplementary information for the labels in square brackets (e.g., [Brazil]). In the non-type specimens, the information was organized as follows: country name in capital and bold letters (e.g., **BRAZIL**), state/department/province name in bold letters (e.g., **Bahía**) and the other information in the original language of the label. Information of the number of examined males (♂) and females (♀) is followed by the text of the label. Finally, the entomological collection they are housed is indicated after each label description in square brackets (e.g., [CEMT]). If there is a semicolon (“;”) after the collection acronym, it means that it has the same data as the previous one, only modified in this information.

**List of abbreviations: ME**: medial endophallite; **SRP**: Superior-right peripheral endophallite; **FLP**: Fronto-lateral peripheral endophallite; **A+SA complex**: Endophallites of the axial and subaxial complex.

## Results and Discussion

### Diagnosis of the* Dichotomius Depressicollis *(Harold, 1867) Species Group

#### Diagnosis

**Fig. 1:**
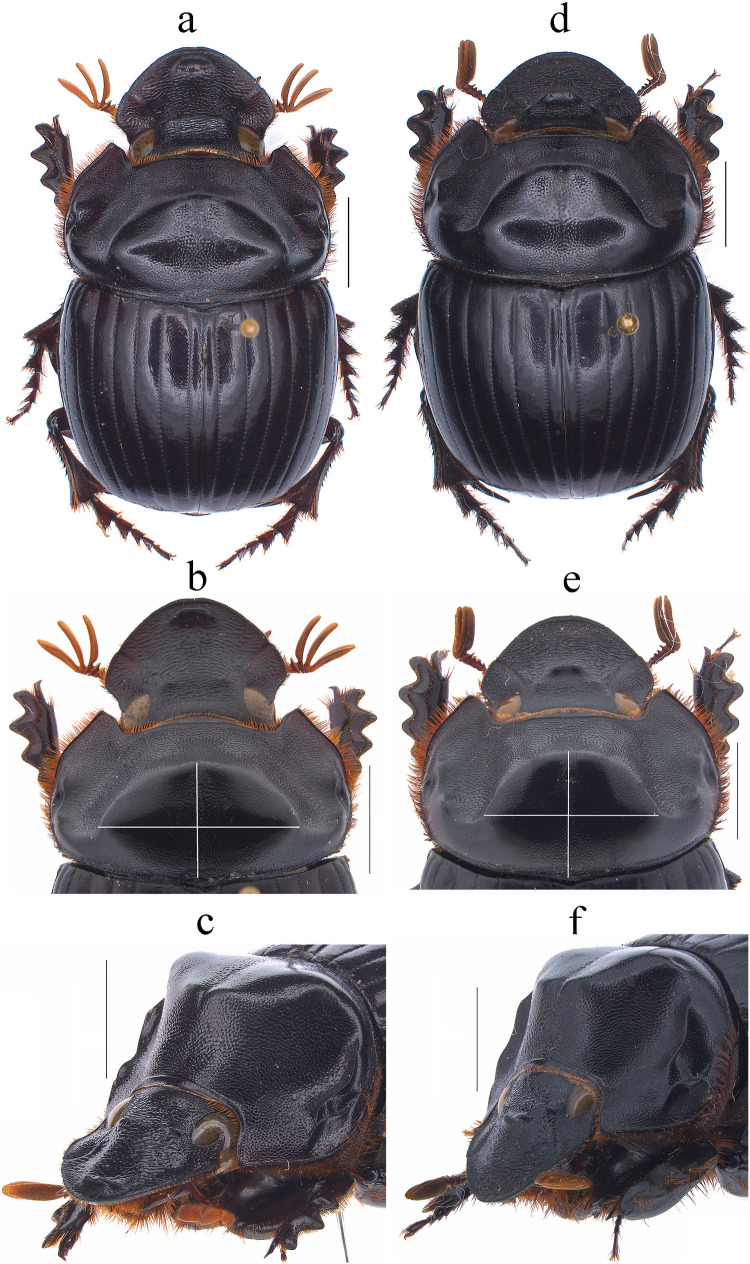
*Dichotomius audinoae* **sp. nov.** Male: **a** Dorsal view. **b** Pronotum with axes. **c** Pronotum in lateraldorsal view. Female: **d** Dorsal view. **e** Pronotum with axes. **f** Pronotum in lateraldorsal view (scale bar 5 mm)

**Fig. 2:**
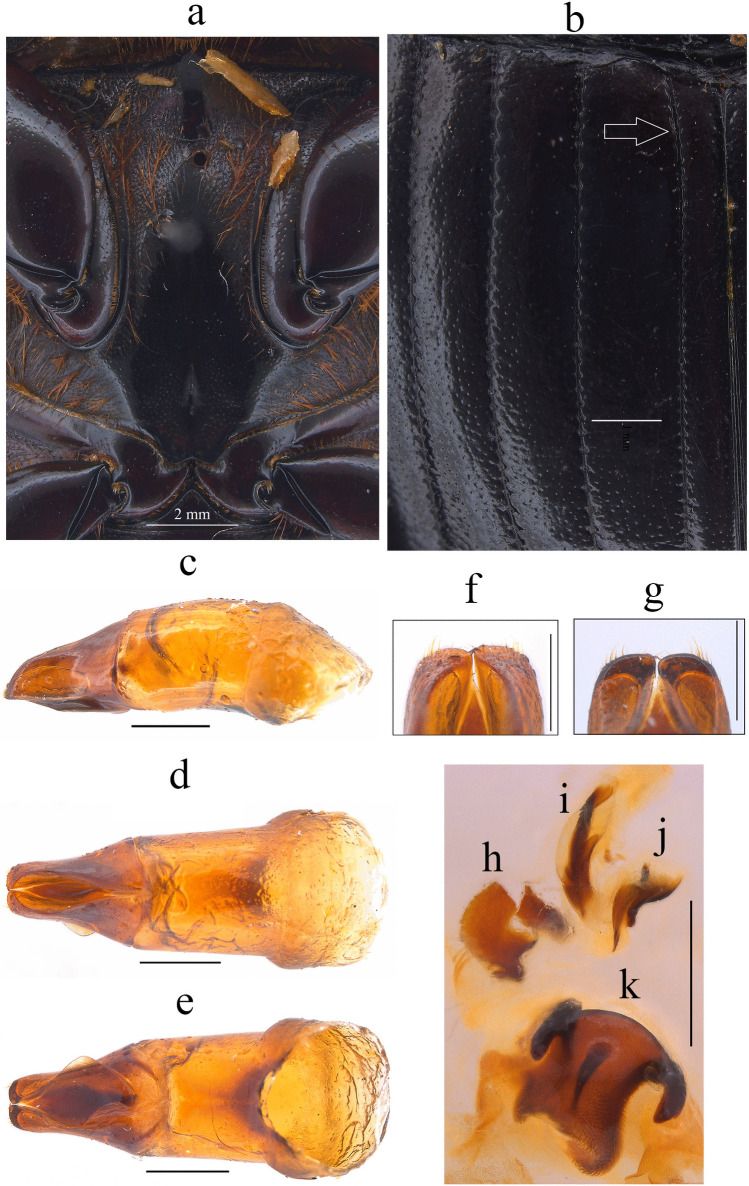
*Dichotomius audinoae* **sp. nov. a** Metaventrite. (scale bar 2 mm). **b** Elytra. Male genitalia: **c** Lateral view, **d** Dorsal view. **e** Ventral view (scale bar 1 mm). **f** Apex of parameres in dorsal view. **g** Apex of parameres in ventral view (scale bar 0.5 mm). Endophallites: **h** SRP. **i** A+SA complex. **j** FLP. **k** ME (scale bar 1 mm)

The species of the *D. depressicollis* species group are recognized by the following combination of characters: (1) Medium-sized to large-sized individuals (17.4–27.3 mm). (2) Head oval as long as it is wide (Figs. 1, 4, 6, 8, 10, 12, 14, 16). (3) Clypeus without clypeal teeth and with strong and well-defined transverse wrinkles (Figs. 1, 4, 6, 8, 10, 12, 14, 16). (4) Ventral clypeal process conical with an obtuse to acute tip. (5) Both sexes with a bidentate head process, in males located in the clypeal region and in females in the fronto-clypeal region (Figs. 1, 4, 6, 8, 10, 12, 14, 16). (6) Protibial spur abruptly angles downward near the apex. (7) Both sexes with an excavated pronotum in the anterior third, with a pronotal disk with a large central lobe as wide as the head, with triangular to rounded shape, the lobe continues on each side to the lateral region where it forms two minor processes over the lateral fovea (Figs. 1, 4, 6, 8, 10, 12, 14, 16). (8) Media-lateral fovea of pronotum elongated and deep, limited by two sinuous subparallel carinae (Figs. 1c, f; 4c, f; 6c, f; 8c, f; 10c, f; 12c, f; 14c, f; 16c, f). (9) Elytral striae bicarinate, shallow to distinctly impressed (Figs. 2b, 5b, 7b, 8b, 11b, 13b, 15b, 17b). (10) Pygidium with an incomplete apical margin and with evident punctures. (11) Aedeagus with a subgenital plate (Fig. 2d, 5d, 7d, 8d, 11d, 13d, 15d, 17d). (12) Parameres subtriangular in lateral view (Fig. 2c, 5c, 7c, 8c, 11c, 13c, 15c, 17c), with short setae at the apex (Fig. 2f, 5f, 7f, 8f, 11f, 13f, 15f, 17f). (13) ME medium to large, subquadrangular with two extensions on the right side and a sclerotized spiny process in the middle, setae present throughout the surface (Fig. 2k, 5k, 7k, 8k, 11k, 13k, 15k, 17k).

**Fig. 3:**
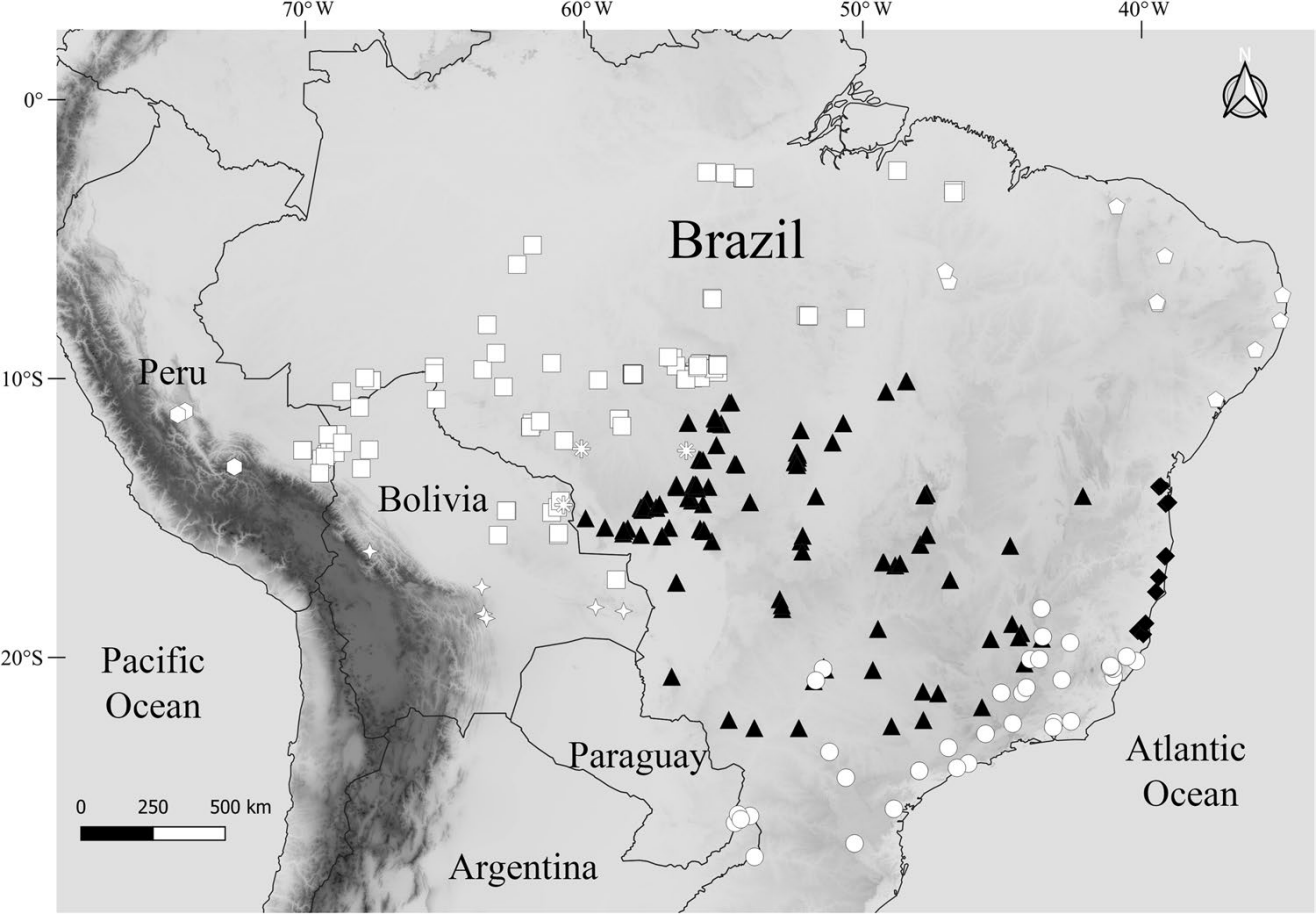
Known distribution of the *Dichotomius depressicollis* species group: *D*. *audinoae* **sp. nov.** (diamonds ♦), *D*. *barberoi* **sp. nov.** (triangles ▲), *D*. *davidedmondsi* **sp. nov.** (stars ✧), *D*. *depressicollis* (circles ●), *D*. *inca* **sp. nov.** (hexagons ⬡), *D*. *melzeri* (squares ■), *D*. *salomaoi* **sp. nov.** (pentagons ⬠), *D*. *zikani* (asterisks *)

**Fig. 4:**
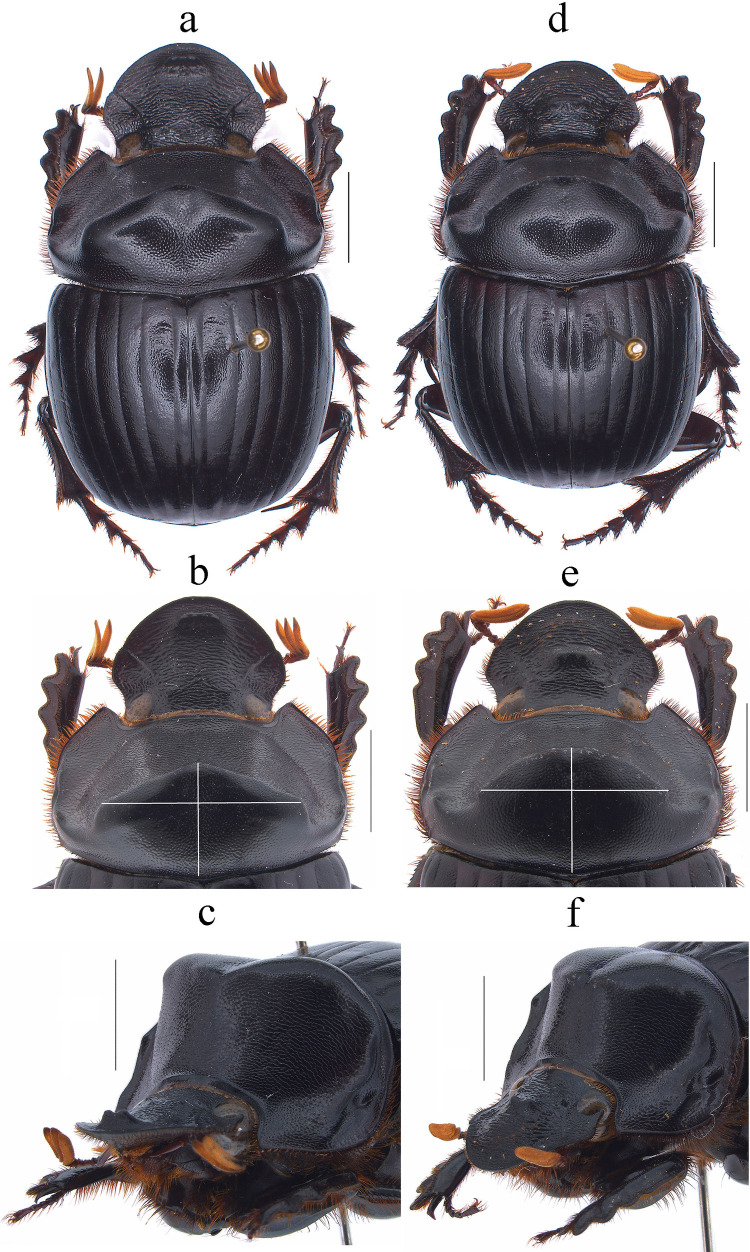
*Dichotomius barberoi* **sp. nov.** Male: **a** Dorsal view. **b** Pronotum with axes. **c** Pronotum in lateraldorsal view. Female: **d** Dorsal view. **e** Pronotum with axes. **f** Pronotum in lateraldorsal view. (scale bar 5 mm)

**Fig. 5:**
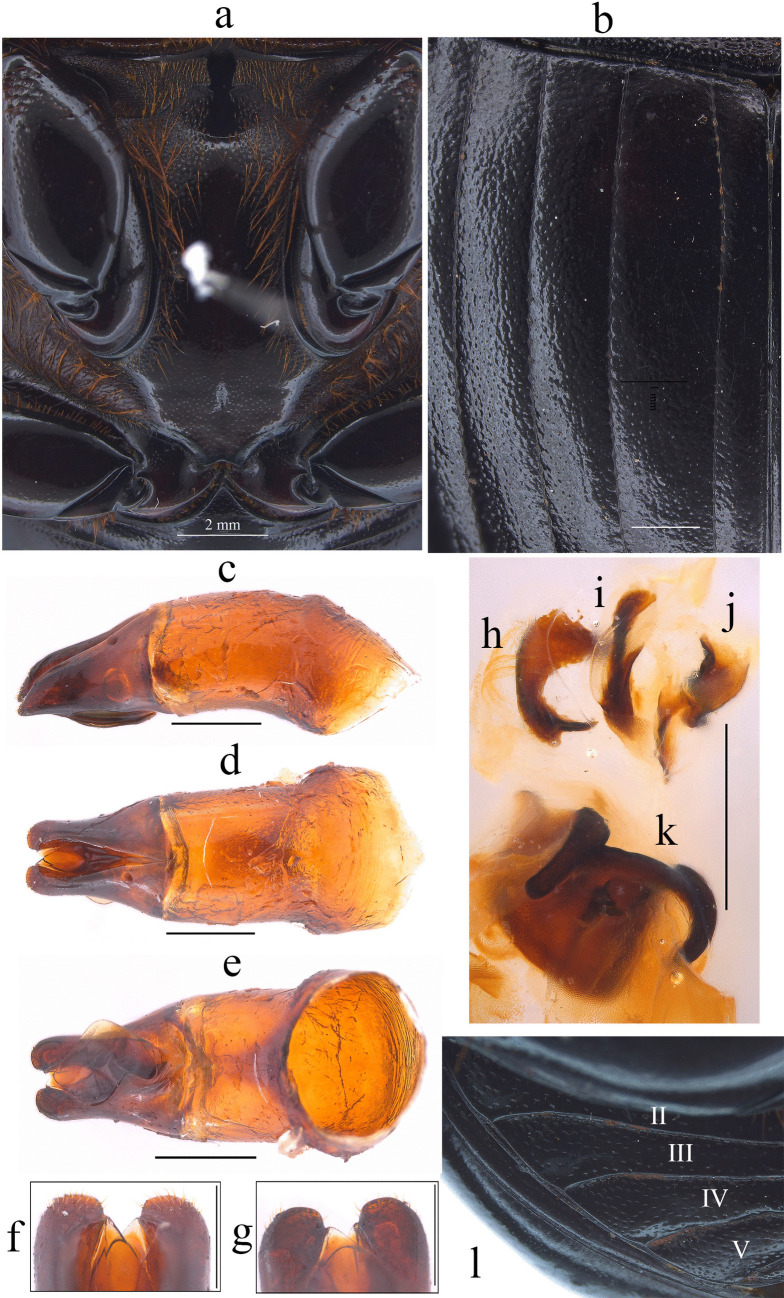
*Dichotomius barberoi* **sp. nov. a** Metaventrite (scale bar 2 mm). **b** Elytra. Male genitalia: **c** Lateral view, **d** Dorsal view. **e** Ventral view (scale bar 1 mm). **f** Apex of parameres in dorsal view. **g** Apex of parameres in ventral view (scale bar 0.5 mm). Endophallites: **h** SRP. **I** A+SA complex. **j** FLP. **k** ME. **l** Abdominal ventrites (scale bar 1 mm)

**Fig. 6:**
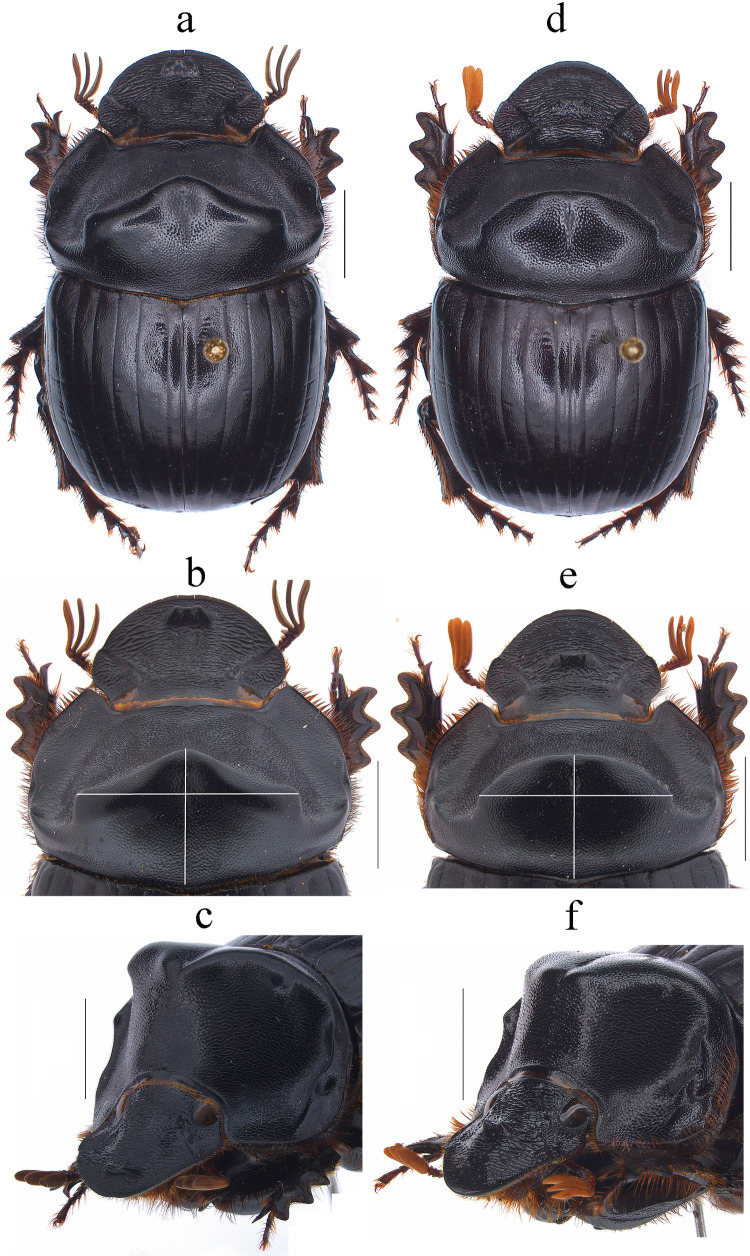
*Dichotomius davidedmondsi* **sp. nov.** Male: **a** dorsal view. **b** Pronotum with axes. **c** Pronotum in lateraldorsal view. Female: **d** Dorsal view. **e** Pronotum with axes. **f** Pronotum in lateraldorsal view (scale bar 5 mm)

**Fig. 7:**
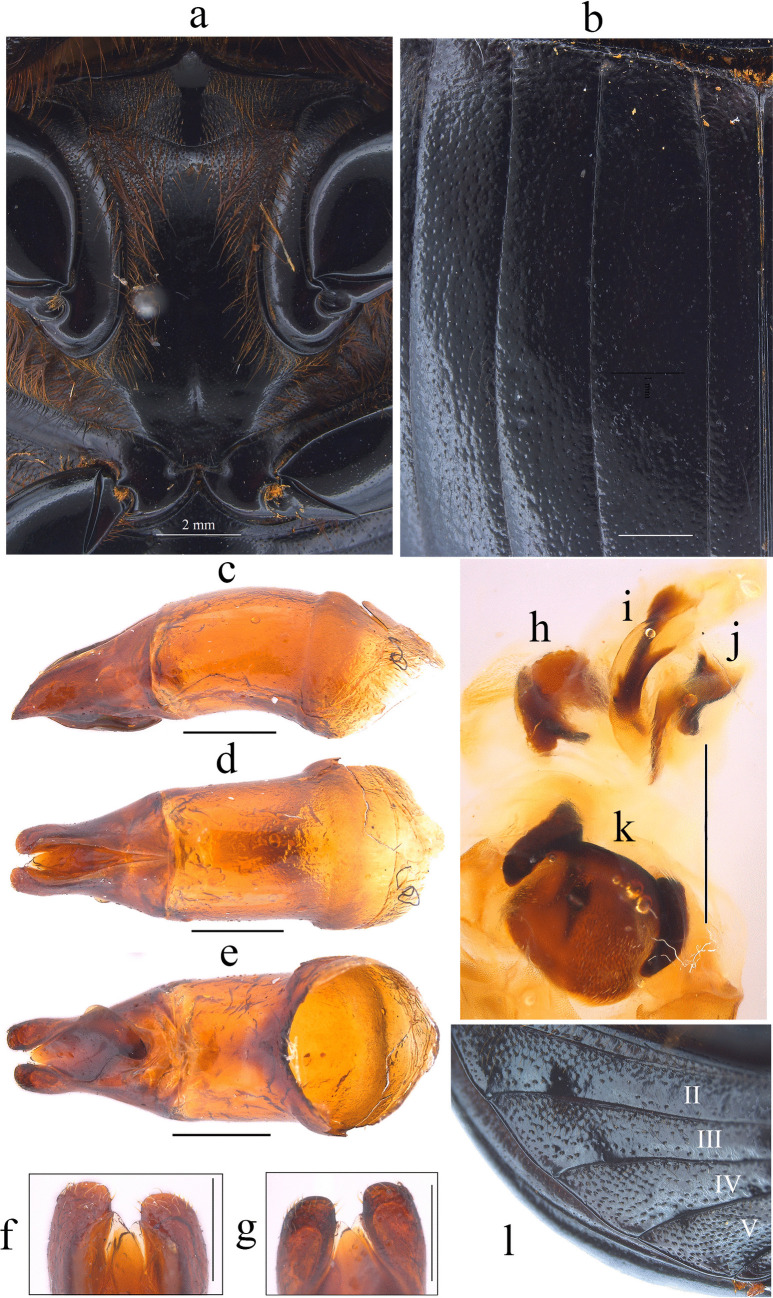
*Dichotomius davidedmondsi* **sp. nov. a** Metaventrite (scale bar 2 mm). **b** Elytra. Male genitalia: **c** Lateral view, **d** Dorsal view. **e** Ventral view (scale bar 1 mm). **f** Apex of parameres in dorsal view. **g** Apex of parameres in ventral view (scale bar 0.5 mm). Endophallites: **h** SRP. **i** A+SA complex. **j** FLP. **k** ME. **l** Abdominal ventrites (scale bar 1 mm)

**Fig. 8:**
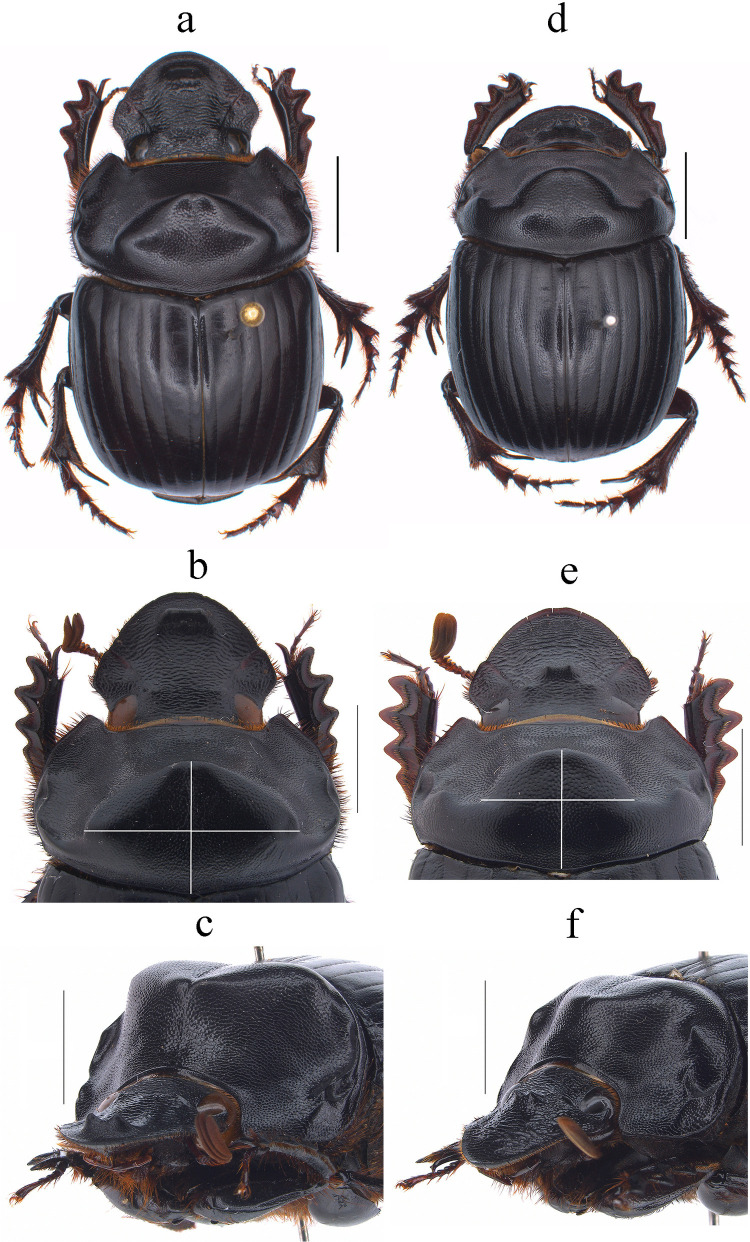
*Dichotomius* *depressicollis*. Male: **a** Dorsal view. **b** Pronotum with axes. **c** Pronotum in lateraldorsal view. Female: **d** Dorsal view. **e** Pronotum with axes. **f** Pronotum in lateraldorsal view (scale bar 5 mm)

**Fig. 9:**
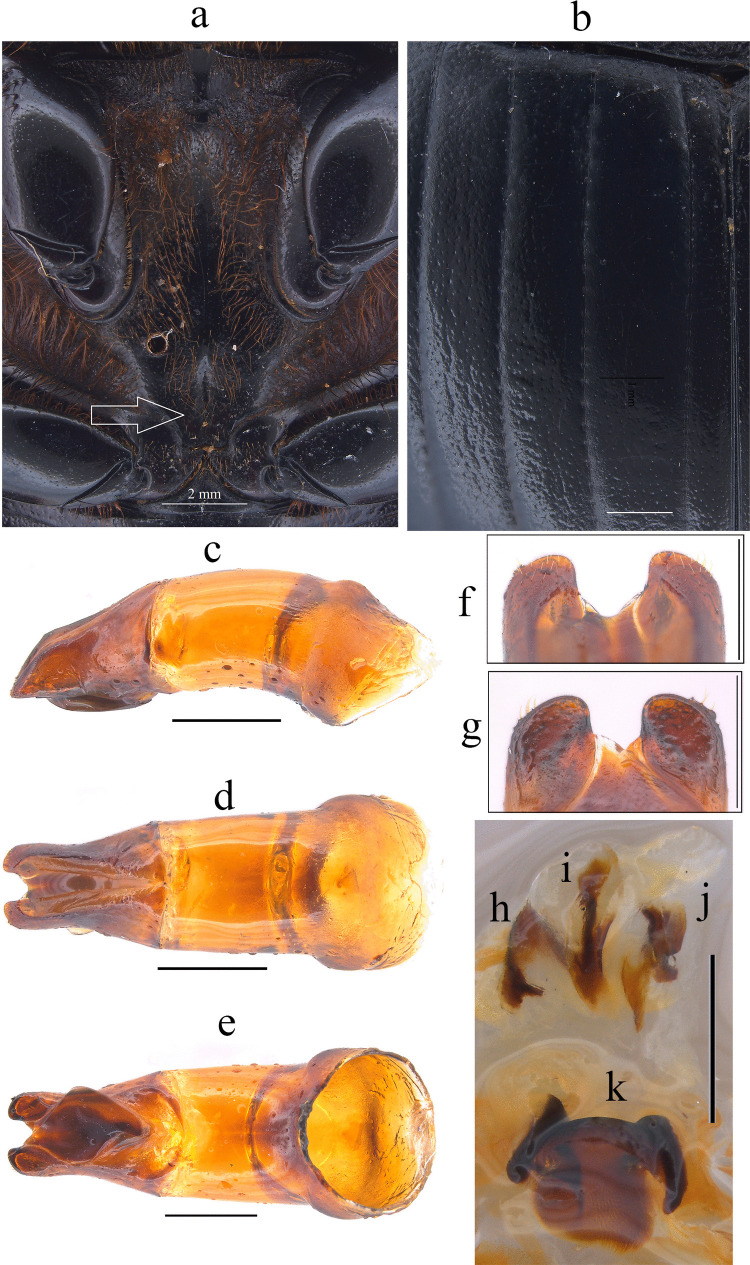
*Dichotomius* *depressicollis*. **a** Metaventrite (scale bar 2 mm). **b** Elytra. Male genitalia: **c** Lateral view, **d** Dorsal view. **e** Ventral view (scale bar 1 mm). **f** Apex of parameres in dorsal view. **g** Apex of parameres in ventral view (scale bar 0.5 mm). Endophallites: **h** SRP. **i** A+SA complex. **j** FLP. **k** ME. **l** Abdominal ventrites (scale bar 1 mm)

**Fig. 10:**
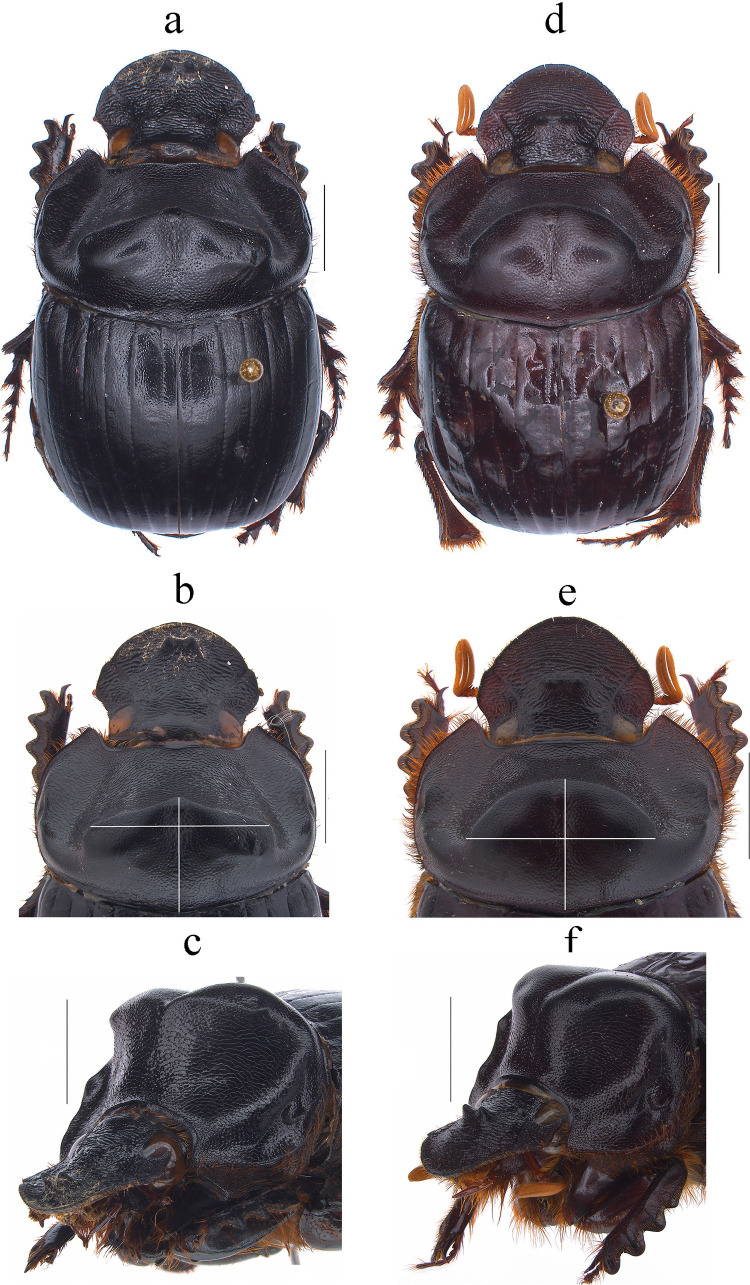
*Dichotomius inca* **sp. nov.** Male: **a** Dorsal view. **b** Pronotum with axes. **c** Pronotum in lateraldorsal view. Female: **d** Dorsal view. **e** Pronotum with axes. **f** Pronotum in lateraldorsal view (scale bar 5 mm)

**Fig. 11:**
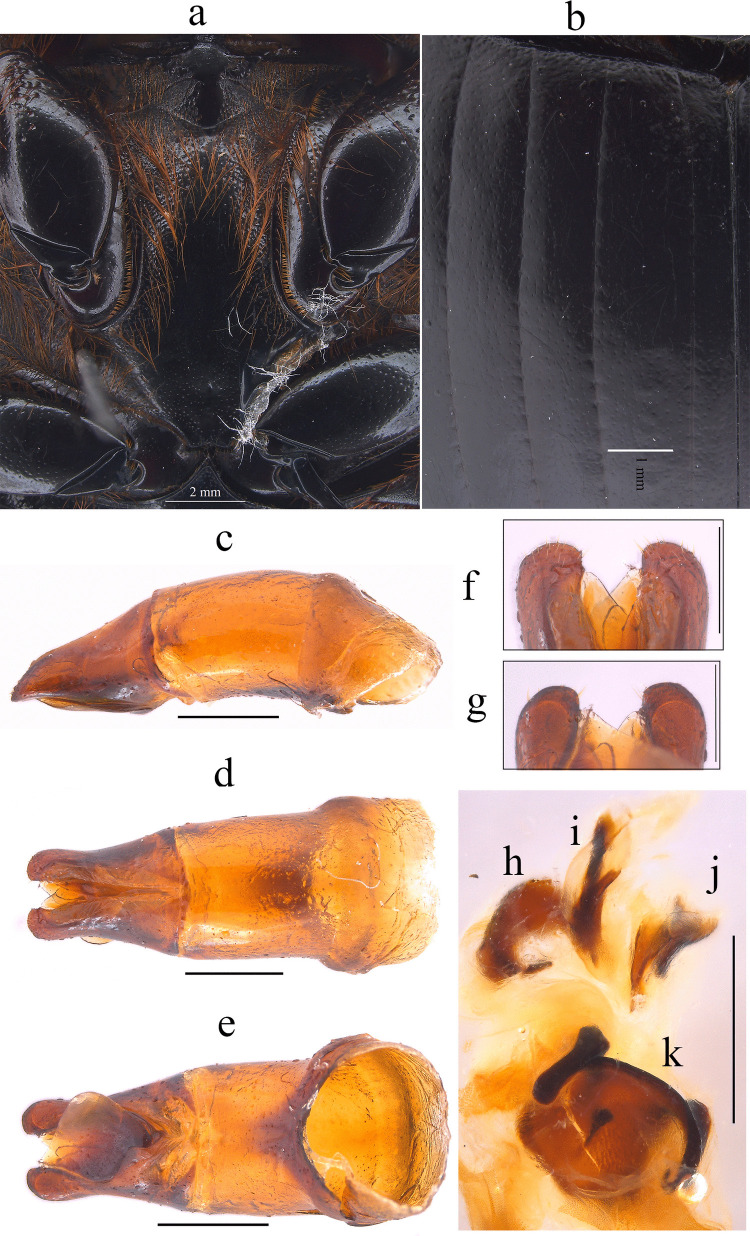
*Dichotomius inca* **sp. nov. a** Metaventrite (scale bar 2 mm). **b** Elytra. Male genitalia: **c** Lateral view. **d** Dorsal view. **e** Ventral view (scale bar 1 mm). **f** Apex of parameres in dorsal view. **g** Apex of parameres in ventral view (scale bar 0.5 mm). Endophallites: **h** SRP. **i** A+SA complex. **j** FLP. **k** ME (scale bar 1 mm)

**Fig. 12:**
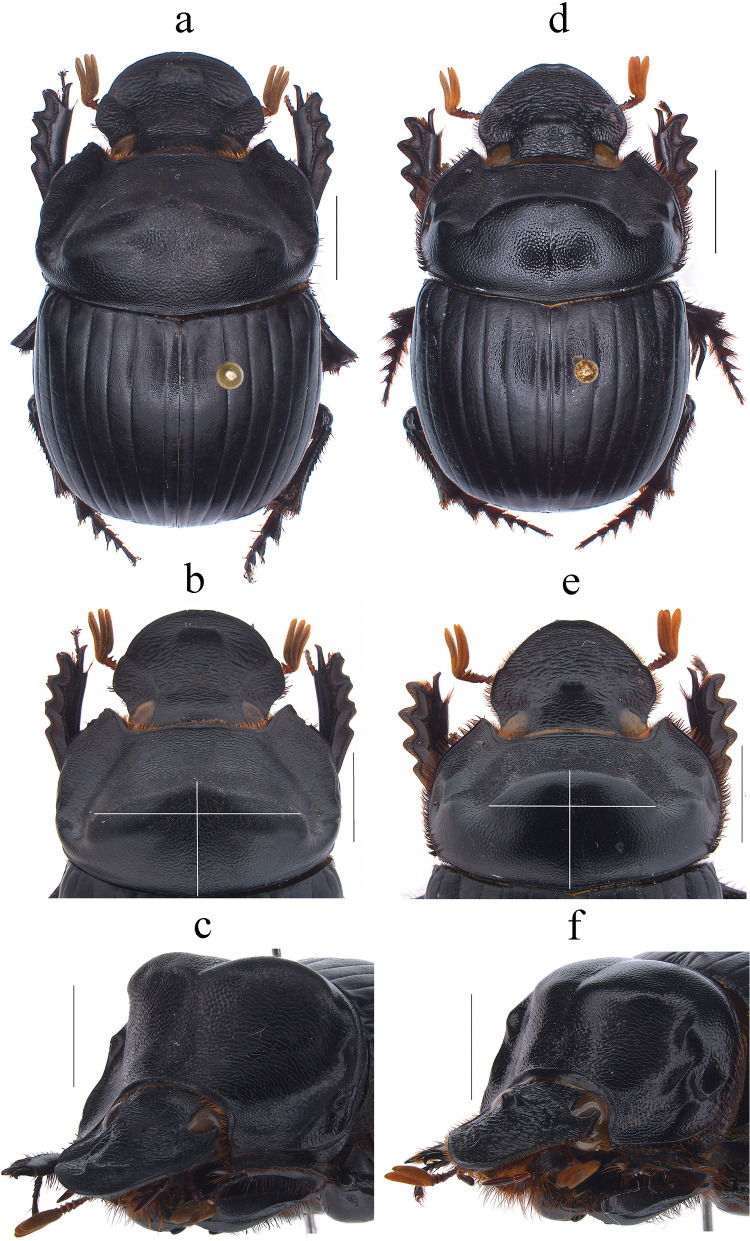
*Dichotomius* *melzeri*. Male: **a** Dorsal view. **b** Pronotum with axes. **c** Pronotum in lateraldorsal view. Female: **d** dorsal view. **e** Pronotum with axes. **f** Pronotum in lateraldorsal view (scale bar 5 mm)

**Fig. 13:**
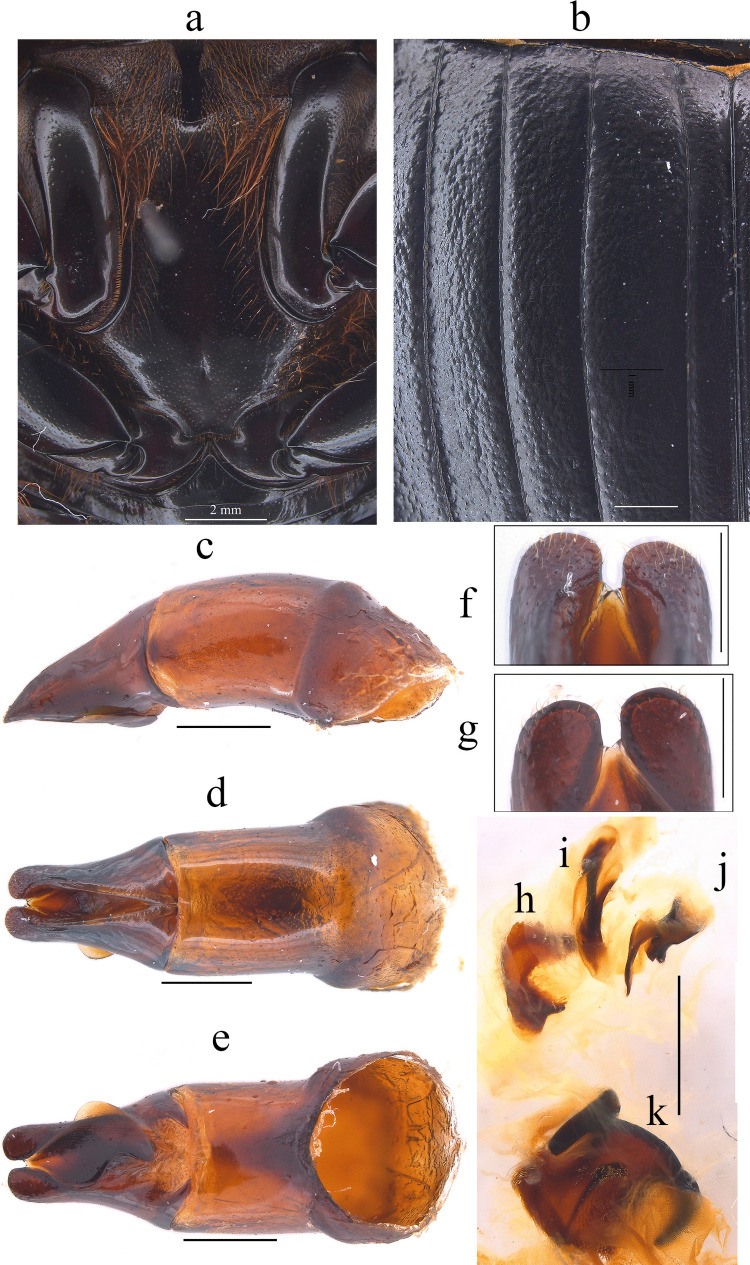
Dichotomius *melzeri*. **a** Metaventrite (scale bar 2 mm). **b** Elytra. Male genitalia: **c** Lateral view. **d** Dorsal view. **e** Ventral view (scale bar 1 mm). **f** Apex of parameres in dorsal view. **g** Apex of parameres in ventral view (scale bar 0.5 mm). Endophallites: **h** SRP. **i** A+SA complex. **j** FLP. **k** ME (scale bar 1 mm)

**Fig. 14:**
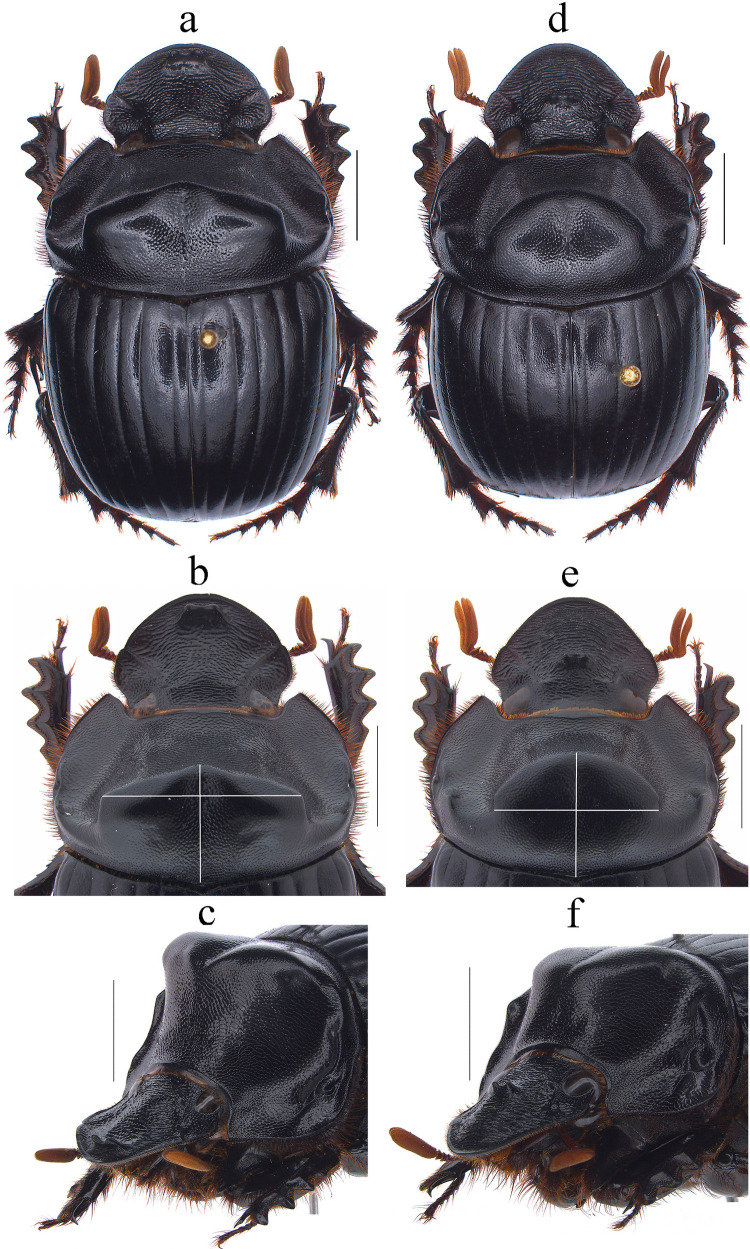
*Dichotomius salomaoi* **sp. nov.** Male: **a** dorsal view. **b** Pronotum with axes. **c** Pronotum in lateraldorsal view. Female: **d** Dorsal view. **e** Pronotum with axes. **f** Pronotum in lateraldorsal view (scale bar 5 mm)

**Fig. 15:**
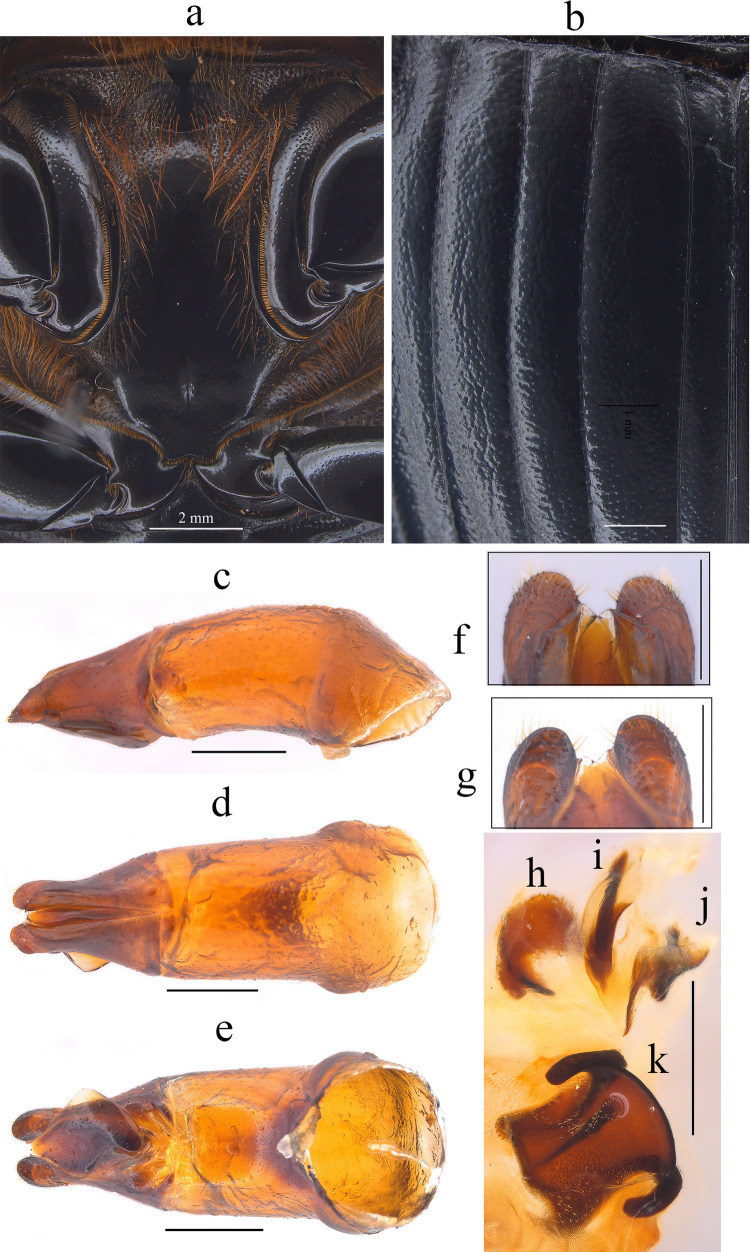
*Dichotomius salomaoi* **sp. nov. a** Metaventrite. (scale bar 2 mm). **b** Elytra. Male genitalia: **c** Lateral view. **d** Dorsal view. **e** Ventral view (scale bar 1 mm). **f** Apex of parameres in dorsal view. **g** Apex of parameres in ventral view (scale bar 0.5 mm). Endophallites: **h** SRP. **i** A+SA complex. **j** FLP. **k** ME (scale bar 1 mm)

**Fig. 16:**
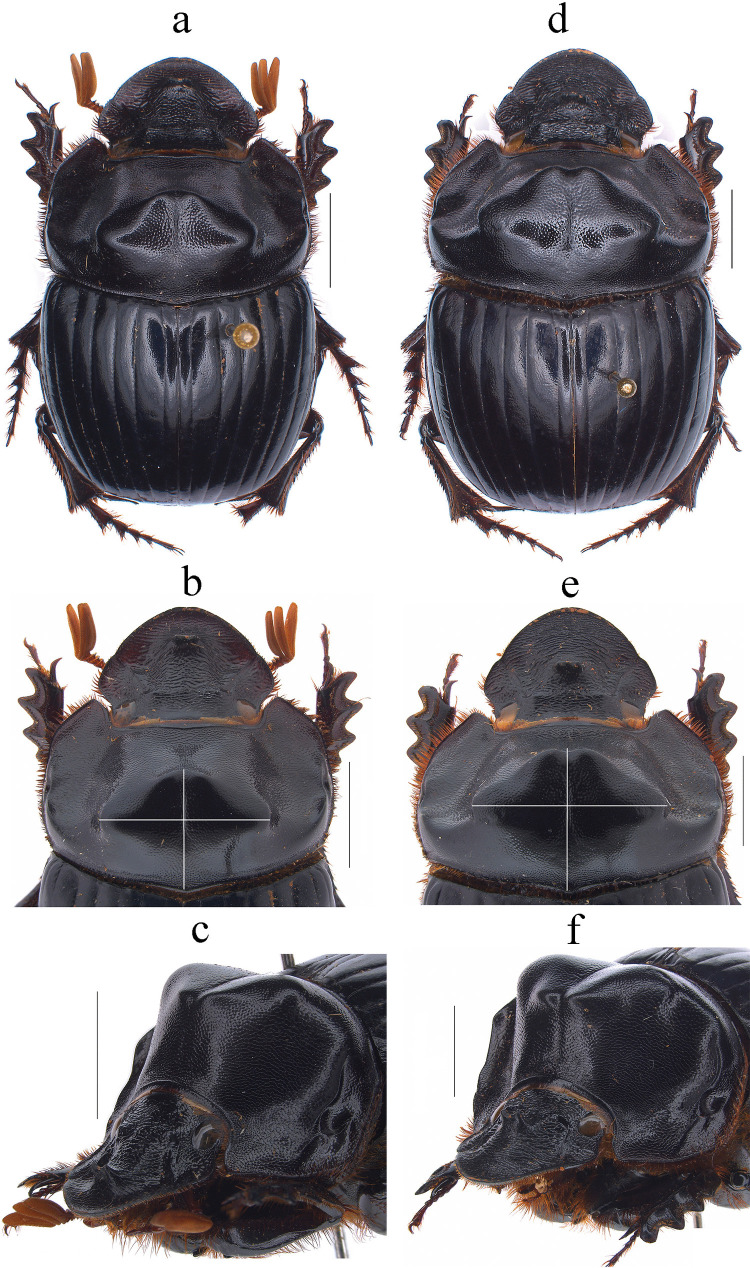
*Dichotomius* *zikani*. **a** Male: **a** Dorsal view. **b** Pronotum with axes. **c** Pronotum in lateraldorsal view. Female: **d.** Dorsal view. **e** Pronotum with axes. **f** Pronotum in lateraldorsal view (scale bar 5 mm)

**Fig. 17:**
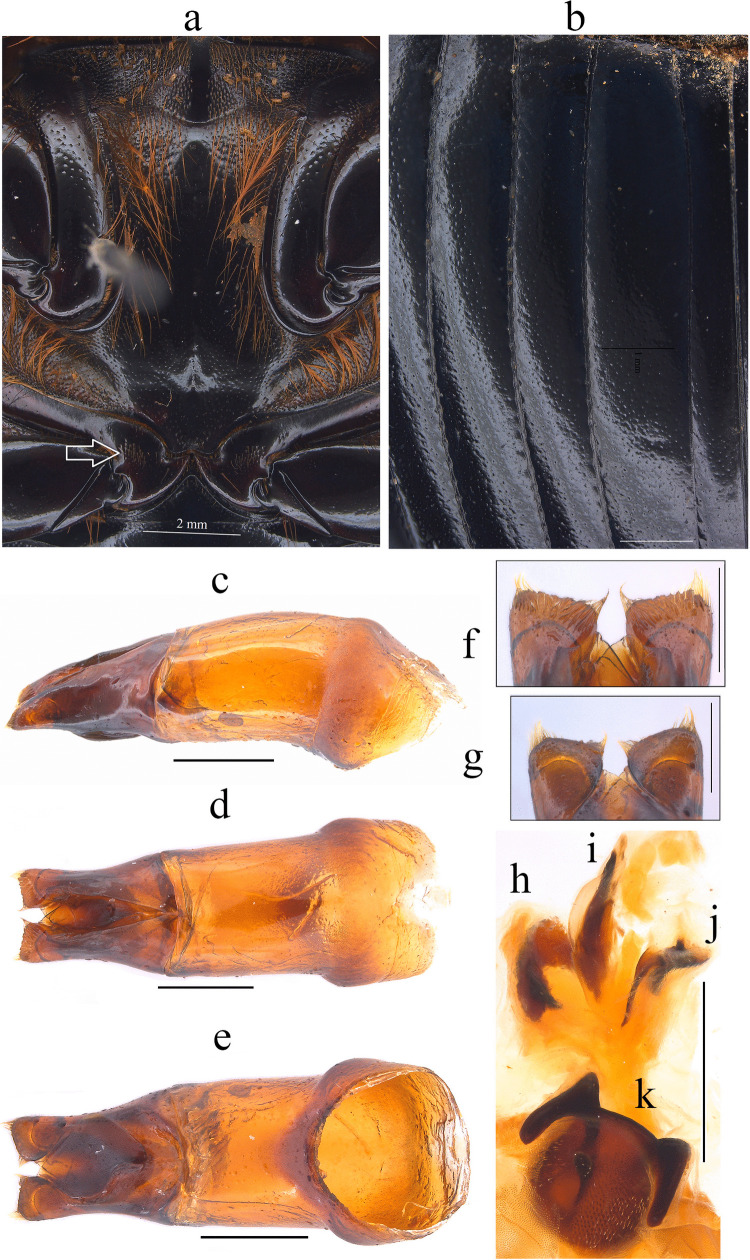
Dichotomius *zikani*. **a** Metaventrite (scale bar 2 mm). **b** Elytra. Male genitalia: **c** Lateral view. **d** Dorsal view. **e** Ventral view (scale bar 1 mm). **f** Apex of parameres in dorsal view. **g** Apex of parameres in ventral view (scale bar 0.5 mm). Endophallites: **h** SRP. **i** A+SA complex. **j** FLP. **k** ME (scale bar 1 mm)

### Description of the* Dichotomius depressicollis* Species Group

#### Body

Length 15.7–28.3 mm; width 8.8–17.4 mm. Black color, some species with blue spots on the elytra, with brown setae on both sides of the head and pronotum.

#### Head

Wider than long. Anterior margin oval, without clypeal teeth in the central region. Anterior margin of clypeus with a shagreened and brilliant flange. Surface with strong parallel wrinkles giving it a wavy appearance. Anterior central region with an upwardly directed, bicuspid process, about 0.2–1.3 mm, ending in a blunt or acute tip. Distinct clypeo-genal suture, with a small protuberance at the base. Genas with a strong corrugated surface.

#### Pronotum

Pronotum wider than long. Curved anterior angles with shiny surface. In lateral view, anterior part of pronotum is diagonal with respect to the back, forming a sharp slope. Anterior surface of short parallel grooves that gives the feeling of being wavy and with microsculpture in these grooves. Pronotum excavated in anterior third or to central region, with pronotal disk with a large central lobe as wide as the head, with triangular to rounded shape, pronotal lobes prolonged on each side to the lateral region where it forms two minor processes over the lateral fovea; with a central longitudinal depression in the middle of process. Media-lateral fovea elongated and deep, limited by two sinuous subparallel carinae. Pronotal disk surface with large, deep punctures variable in density among species. Pronotal process slightly poorly developed in minor specimens. **Hypomeron**: With ocellate and setose punctures, separated once diameter and joined in some sites. **Propleuron**: With setose punctures in the lateral areas and smooth in the central area. **Prosternum**: Shagreened with dense, setose punctures. **Mesoventrite**: Shagreened with setose punctures with long and soft setae. **Mesanepisternum**: Ocellate, setose punctures separated by less than one-half the diameter. **Metaventrite**: Surface with setae variable in distribution and density among species. With strong carina that extends from 1/3 of metaventrite to the posterior region, ending in deep fovea with shiny and glabrous surface near to carina. Lateral edges of anterior region shagreened with dense setose punctures with long setae; posterior edges with or without setose punctures, varying in each species. **Metanepisternum**: with equal area to the side of the mesanepisternum. **Elytra**: Striae bicarinate, with medium to large ocellate punctures along its entire length, separated about two to three times their diameter.

#### Legs

Profemur in ventral view with long setae on all extensions of the anterior and posterior margin. Shagreened surface with deep and large punctures and with two to three rows of setose punctures with long setae. Protibiae with four teeth, with shiny and distinct margin. Anterior margin of protibia obliquely truncate, inner and apical angles finely acuminate, with straight setae on the apex. Anterior protibial carinae internally bordered by a row of deep punctures, each with a long setae. Dorsal surface on inner side with deep and large punctures. Protibial spur abruptly curved distally, with acute apex. Five tarsomeres, the first and last larger than 2–4 joined. Mesofemur with spaced long setae on the anterior margin, shiny to shagreened surface, with elongate and sparse punctures, and dense deep and large setose punctures in the apical half. Mesotibia with 8–10 small spines on outer-lateral side, each with an erect setae; basitarsomere as long as tarsomeres 2–4 joined. Metafemur with spaced long setae on the anterior margin, shiny to shagreened surface, with elongate and sparse punctures and a row of 8–12 setose punctures in the apical region; metatibia with 10–12 small spines on outer-lateral side, each with an erect setae; basitarsomere as long as tarsomeres 2–4 joined. Metatibial spur with truncate apex.

#### Abdomen

Shagreened ventrites with two or three rows of ocellate points on anterior area, becoming denser towards the side; fifth ventrite with more punctures laterally. Sixth ventrite narrow medially, with punctures laterally. **Pygidium**: with incomplete apical margin. Shinny surface, with evident elongate punctures separated by twice its diameter.

#### Aedeagus

Parameres triangular in lateral view, more sclerotized in the apical region, lower basal part with an acute process. In dorsal view, symmetrical or asymmetrical parameres with a widened base, decreasing in thickness towards apical region ending in a blunt tip with short setae; 1/3 from apical region with broad border. The inner border is convex from the middle to the apical region. Left paramere with subgenital plate partially visible. In ventral view, parameres broad at basal region with an acute prolongation; apical region narrow with blunt apex. A subgenital plate present, broad covering the inner margin of the left paramere is not visible; on the right side it protrudes beyond the paramere. **Endophallites**: ME large and subquadrangular with two well sclerotized processes on the right lateral side and a sclerotized process with spike shape in the middle, the surface covered with bristles along its entire length, and on the left side it presents a membranous process with long bristles. A + SA complex is sclerotized in the central area surrounded by a semi-sclerotized membrane. SRP has a “C” shape, wide. FLP has an “N” shape.

#### Females

Length 17–27,1 mm, width 10.9–17.4 mm. It differs from males for: bicuspid process located in the center of head from 0.2–1.3 mm, directed upwards; in some individuals the tip is flat. In some species the central process of pronotum is rounded at the anterior region. Abdomen, with sixth ventrite not compressed toward middle region.

### Identification Key to the Species of the *Dichotomius depressicollis* Species Group

1. Cephalic process in clypeal region. Sixth abdominal ventrite narrow medially (males)…2.
  Cephalic process in fronto-clypeal region. Sixth abdominal ventrite broadened medially (females)…9.
2. Postero-central region of metaventrite near to the insertion of metacoxa with denser and long setae (Fig. 9a). Atlantic forest and transition to Cerrado biome (Fig. 3 circles)… ***Dichotomius depressicollis*** **(Harold, **1867**)**.
  Postero-central region of metaventrite near to the insertion of metacoxa with few short or absent setae (Figs. 2a, 5a, 7a, 11a, 13a, 15a, 17a)…3.
3. Elytra with striae regularly interrupted by deep punctures, giving an impression of waving (evident near to the base of the 1 st striae) (Fig. 2b). Atlantic forest northern of Rio Doce (Fig. 3 diamonds)… ***Dichotomius audinoae*** **sp. nov.**
  Elytra with striae with shallow punctures not giving an impression of waving (Figs. 5b, 7b, 11b, 13b, 15b, 17b)…4.
4. Pronotal process with the axes formed between their tips forming a plus sign (+). Pronotal process in dorsal view with two well-defined central tips (Fig. 16a–b). Metatrochanter with setose punctures with long setae (Fig. 17a). Bolivia (Santa Cruz) and Brazil (Mato Grosso and Rondônia) (Fig. 3 asterisks)… ***Dichotomius zikani*** **(Luederwaldt, **1922**)**.
  Pronotal process with axes formed between their tips forming a cross (†) (Figs. 4b, 6b, 10b, 12b, 14b). Metatrochanter without setose punctures (Figs. 5a, 7a, 11a, 13a, 15a)…5.
5. Pronotal process rounded and wide in central region (Fig. 12a–b).Metaventrite with dense setae on the anterior surface reaching to the central region of the mesocoxae, some individuals may have a few setae or a line of setae, but never abundant (Fig. 13a). Surface of pronotal process with large circular to elongate punctures spaced less than one times their size and joined in some places, giving the appearance of a wavy surface (Fig. 12a–b). Amazon of Bolivia, Brazil, and Peru (Fig. 3 squares)… ***Dichotomius melzeri*** **(Luederwaldt, **1922**)**.
  Pronotal process in the central region with a tip giving the appearance of a triangle (Figs. 4b, 6b, 10b, 14b). Metaventrite with dense and continuous setae from anterior lobe to lateral sides near mesocoxal insertion (Figs. 5a, 7a, 11a, 15a). Surface of pronotal process with small circular punctures, separated and visible at lowest magnification 10X (Figs. 4b, 6b, 10b, 14b)…6.
6. Surface of interstriae with smooth appearance principally in the apical region; with small punctures spaced twice their diameter, decreasing in depth towards the apical region (Fig. 11b). High altitudes (> 692 m) of Peru (Fig. 3 hexagons)… ***Dichotomius inca*** **sp. nov.**
  Surface of interstriae with wavy appearance in all extensions, with deep and distinct punctures (Figs. 5b, 7b, 15b). Low altitudes (< 300 m) of Bolivia and Brazil (Fig. 3)…0.7
7. Lateral areas of first to fifth abdominal ventrite with small setae (Fig. 7l). Evident asymmetric parameres, with the right one shorter (Fig. 7d–g). Chiquitano dry forest of Bolivia (Fig. 3 starts)… ***Dichotomius davidedmondsi*** **sp. nov.**
  Lateral areas of first to fifth abdominal ventrite without setae (Fig. 5l). Symmetric parameres or not evident asymmetric (Fig. 5d–g, 16g–d). Brazil…8.
8. Interstriae surface with wrinkles in all extensions, with small and deep punctures (Fig. 15b). Ventral surface of apex of parameres with evident setae (Fig. 16g). Caatinga and subxerophytic regions of northeastern Brazil (Fig. 3 pentagons)… ***Dichotomius salomaoi*** **sp. nov.**
  Interstriae surface with evident wrinkles in the basal area and less evident in the apical area, with small and soft punctures (Fig. 5b). Ventral surface of apex of parameres without setae (Fig. 5g). Brazilian Cerrado (Fig. 3 triangles)… ***Dichotomius barberoi*** **sp. nov.**
9. Postero-central region of metaventrite near to insertion of metacoxa with denser and long setae (Fig. 9a). Atlantic forest and transition to Cerrado biome (Fig. 3 circles)… ***Dichotomius depressicollis*** **(Harold, **1867**)**.
  Postero-central region of metaventrite near to insertion of metacoxa with few short or absent setae (Figs. 2a, 5a, 7a, 11a, 13a, 151, 17a)…10.
10. Elytra with striae regularly interrupted by deep punctures, giving an impression of waving (evident near to the base of the 1 st striae) (Fig. 2b). Atlantic forest northern of Rio Doce (Fig. 3 diamonds)… ***Dichotomius audinoae*** **sp. nov.**
  Elytra with striae with shallow punctures (Figs. 5b, 7b, 11b, 13b, 15b, 17b)…11.
11. Pronotal process with two well-defined central points and two lateral points (Fig. 16d–e). Metatrochanter with setose punctures with long setae (Fig. 17a). Bolivia (Santa Cruz) and Brazil (Mato Grosso and Rondônia) (Fig. 3 asterisks)… ***Dichotomius zikani*** **(Luederwaldt, **1922**)**.
  Pronotal process without two well-defined central points (Figs. 4d, 6d, 10d, 12d, 14d). Metatrochanter without setose punctures (Figs. 5a, 7a, 11a, 13a, 15a)…12.
12. Pronotal process with the axes formed between their tips forming a plus sign (+) (Figs. 10e, 12e, 14e)…13.
  Pronotal process with the axes formed between their tips forming a cross (†) (Figs. 4e, 6e)…15.
13. Pronotal process rounded and wide in central region. Metaventrite with dense setae on the anterior surface reaching to the central region of the mesocoxae; some individuals may have a few setae or a line of setae, but never abundant. Surface of pronotal process with large circular to elongate punctures spaced less than one times their size and joined in some places, giving the appearance of a wavy surface (Fig. 12d–e). Amazon of Bolivia, Brazil, and Peru (Fig. 3 squares)… ***Dichotomius melzeri*** **(Luederwaldt, **1922**)**.
  Pronotal process rounded and slender in central region. Metaventrite with dense and continuous setae from anterior lobe to lateral sides near mesocoxal insertion (Figs. 5a, 7a, 11a, 15a). Surface of pronotal process with small circular punctures, separated at lowest magnification (Fig. 10d–e, 14d–e)…14.
14. Surface of interstriae with smooth appearance principally in the apical region; with small punctures spaced twice their diameter, decreasing in depth towards the apical region (Fig. 11b). High altitudes (> 692 m) of Peru (Fig. 3 hexagons)… ***Dichotomius inca*** **sp. nov.**
  Surface of interstriae with wavy appearance in all extensions, with deep and distinct punctures (Fig. 15b). Caatinga and subxerophytic regions of northeastern Brazil (Fig. 3 pentagons)… ***Dichotomius salomaoi*** **sp. nov.**
15. Lateral edges areas of first to fifth abdominal ventrite with small setae (Fig. 7l). Chiquitano dry forest of Bolivia (Fig. 3 starts)… ***Dichotomius davidedmondsi*** **sp. nov.**
  Lateral areas of first to fifth abdominal ventrite without setae (Fig. 5l). Brazilian Cerrado (Fig. 3 triangles)… ***Dichotomius barberoi*** **sp. nov.**

1. ***Dichotomius (Dichotomius) audinoae*** **Arias-Buriticá & Vaz-de-Mello sp. Nov**

http://zoobank.org/urn:lsid:zoobank.org:act:8EF075C6-552D-4DA8-BEB1-D8B9C0F295CF

(Figs. 1a–f, 2a–k, 3 diamonds)

#### Type Material

**Holotype**: 1♂. Labels: 1: {printed text on white label} BRASIL, Bahía, Igrapiúna Adeilton. 13°51′12.1″S 39°18′55.8″W—18-ii-2016 human dung # 3 PPLopes/2: {printed and handwritten text on red label} **HOLOTYPE** *Dichotomius audinoae* sp. nov. Arias-Buriticá & Vaz-de-Mello, 2026 [CEMT]./3: {printed text on white label with black margins} QR code CEMT CUIABÁ 00115298. **Paratypes**: same data of holotype. 1**♂**. [CEMT]. **BRAZIL**: **Bahía**: Caravelas Mussununga 17°38′52.1″S 39°29′35.3″W 51 m 14–17-IX-2014 Vargas T. 1♂. [CEMT]. Pirai do Norte Waldemar 13°52′55″S 39°22′53.6″W 18-II-2016 human dung Lopes PP. 1♂. [CEMT]. Porto Seguro VII-1993 Grossi E. 1♂ 1♀. [CEMT]. Porto Seguro R[eserva] P[articular do] P[atrimonio] N[atural Estação] Veracel XI-2004 Pitfall c/hum. Faec. Louzada JNC. 1♂ 1♀. [CEMT]; same data but, [19-XI-2004]. 1♀. [CEMT]. Porto Seguro R[eserva] P[articular do] P[atrimonio] N[atural Estação] Veracel 16°21′31″S 39°7′41″W 85 m secondary forest 13-V-2012 human feces Audino LD. 1♀. [CEMT]. Prado Parque Nacional Descobrimento 17°7′30.6″S 39°23′17.2″W 30-XI-2014 Pitfall Fez. hum. Costa CMQ. et al. 3♀. [CEMT]. Uruçuca Parque Estadual Serra do Conduru 14°29′42.6″S 39°8′17.2″W 27-XI-2014 Pitfall Fez. hum. Costa CMQ. et al. 2♂. [CEMT]. Uruçuca 14°26′24″S 39°02′19″W Atlantic forest Pitfall hum-dung 08–11-XII-2022 Ferreira JVA. 1♂. [CEMT]. **Espírito Santo**: Linhares Res[erva] Nat[ural] Vale PPBIO 19°10′10″S 39°56′43″W 13-V-2016 pitfall human feces Cassar F. 4♂ 3♀. [CEMT]. Sooretama 19°3′15″S 40°8′52.3″W 13-IV-2014 Pitfall Fez. Hum. Costa CMQ. et al. 1♂. [CEMT]. All paratypes have a second label: 2: {printed text on yellow label with black margins} *Dichotomius audinoae* sp. nov. Arias-Buriticá & Vaz-de-Mello, 2026 [♂ or ♀] PARATYPE.

#### Diagnosis

This species can be distinguished from the other members of the *D*. *depressicollis* species group by the following combination of characters: Both sexes with pronotal process with the axes formed between their tips forming a plus sign (+) (Fig. 1), elytra with striae distinctly impressed, shagreened, and regularly interrupted by deep punctures, giving an impression of waving (evident near to the base of the 1 st striae) (Fig. 2b), metaventrite near to the insertion of metacoxa without setae (Fig. 2a), metatrocanther without setae (Fig. 2a) morphology of the male genital organ as in Fig. 2c-g, and shape of endophallites (Fig. 2h-k).

#### Etymology

This species is named in honor of Dra. Lívia Dorneles Audino in recognition of her contribution to ecology and conservation of Atlantic Forest dung beetles.

#### Description

**Male**: Length 24.6 mm; width 15.3 mm (Fig. 1a). **Pronotum**: On the top of the pronotum in the central area, that reaches the anterior third, has a central process as long as wide that forms a plus sign (+) with its axes, with three pronounced points and the central one with two lobes (Fig. 1a, b). Surface of the pronotal disk with large and deep punctures presenting microsculpture inside (seen at high magnification) separated less than their diameter, dense in the posterocentral region of the pronotal disk and decreasing towards the apex of the pronotal protuberances (Fig. 1a–c). **Metaventrite**: Postero-central region of metaventrite near to insertion of metacoxa without setae (Fig. 2a). **Elytra**: Striae bicarinate, with striae distinctly impressed, shagreened, and regularly interrupted by deep punctures, giving an impression of waving evident near to the base of the 1 st striae (Fig. 2b). Interstriae with shiny surface, with some shallow wrinkles near to the base; with large and shallow punctures, separated by three times their diameter and visible under low magnification (Fig. 2b). **Abdomen**: third to fifth ventrites without lateral setae.

#### Aedeagus

Parameres triangular with wide apex in lateral view (Fig. 2c). In dorsal view, parameres symmetrical; with a widened base, decreasing in thickness towards apical region ending in a rounded tip with short setae, longer on the sides (Fig. 2f). Left paramere with subgenital plate partially visible (Fig. 2d). In ventral view, parameres are broad at basal region with an acute prolongation; apical region is narrow with truncate apex without setae (Fig. 2g). A subgenital plate is present, broad, protruding beyond the paramere on the right side (Fig. 2e). **Endophallites**: ME large and subquadrangular with two well sclerotized processes on the right lateral side, the surface covered with bristles along its entire length, and on the left side it presents a membranous process with long bristles and with the spine toward the right edge (Fig. 2k). A+SA complex is sclerotized in the central area surrounded by a semi-sclerotized membrane (Fig. 2i). SRP has a “C” shape with wide base (Fig. 2h). FLP has an “N” shape (Fig. 2j).

#### Variation

In males, length varies from 20.3 to 27.7 mm and the width from 12.8–16.5 mm; the cephalic process varies in length from 0.2 to 1.3 mm. Some specimens have blue spots on the elytra. **Females**: the length varies from 20.2 to 25.9 mm and the width from 13.1 to 16.9 mm; the cephalic process varies in length from 0.2 to 0.8 mm. In the pronotum, the central process is rounded at the anterior margin (Fig. 2d and e).

#### Distribution and Ecology

Atlantic Forest of Brazil, northern of the Rio Doce, in the states of Bahia and Espírito Santo (Fig. 3). It was collected in February, April, May, July, September, and November, from 60 to 290 m.a.s.l., using pitfall traps baited with human dung.

2. ***Dichotomius (Dichotomius) barberoi*** **Arias-Buriticá & Vaz-de-Mello sp. nov**

http://zoobank.org/urn:lsid:zoobank.org:act:8603D801-F338-4A83-9E44-4948450BB9C1

(Figs. 4a–f, 5a–l, 3 triangles)

#### Type Material

**Holotype**:1♂. Labels: 1: {printed text on white label}: Brazil: Mato Grosso: Tangará da Serra Sitio Boa Vista 14°36′42″S 57°50′12″W 328 m Floresta semidecidual 09–11-II-2012 Pitfall hum-pig dung Silva RJ./2: {printed and handwritten text on red label} **HOLOTYPE** *Dichotomius barberoi* sp. nov. Arias-Buriticá & Vaz-de-Mello, 2026 [CEMT]./3: {printed text on white label with black margins} QR code CEMT CUIABÁ 00115125. **Paratypes**: same data of holotype. 1♂ [CEMT]. **BRAZIL**: **Distrito Federal**: Brasília F[azenda] Á[guas] L[impa]-Un[iversidade de] Brasília 15°56′25.1″S 47°56′22.2″W cerrado mata de galleria 27-XII-2013 Pitfall-fezes Bernardes T. 4♂ 4♀. [CEMT]. Brasília Fazenda Agua Limpa 15°57′21.9 S 47°57′3.4″W cerrado 10-XII-2017 Pitfall fezes porco Ferreira Y. 1♂. [CEMT]. Planaltina Embrapa 15°35′47.8″S 47°42′20.1″W cerrado 04-XII-2017 Pitfall fezes porco Ferreira Y. 1♀. [CEMT]. Planaltina Embrapa CPAC 15°36′24.7″S 47°42′45.9″W 1025 m 22-XI-2013 interceptação Oliveira CM. 1♂. [CEMT]; same data but, [09-XII-2013]. 1♂. [CEMT]. **Goiás**: Bom Jardim de Goiás galería II-1997 Vaz-de-Mello. 2♂. [CEMT]. Chapada dos Veadeiros 14°6′16.83″S 47°42′24.04″W 06-XII-2015 Armad[ilha de] Queda Rocha MVC. 5♂ 6♀. [CEMT]; same data but, [14°7′8.26″S 47°43′50.11″W]. 1♀. [CEMT]; same data but, [14°6′16.83″S 47°42′24.04″W]. 1♀. [CEMT]. São Jorge Chapada dos Veadeiros 14°9′42″S 47°47′24″W I-2011 hum[an] fec[es] Nunes R. 1♂. [CEMT]. Chapadão do Céu Parque Nacional [das] Emas 18°15′49″S 52°53′30″W 11-XI-2018 ligth Hufnagel L. 1♀. [CEMT]. Mineiros P[arque] N[acional das] Emas 17-IV-2004 Albertoni SM. 1♀. [CEMT]. Mineiros P[arque] N[acional das] Emas 17°54′31″S 52°58′59″W 823mosl 15-III-2011 hum[an] dung Souza MF. 1♂. [CEMT]. Goiânia U[niversidade] F[ederal de] Goiás Agronomia 16°35′32″S 49°17′3″W 738 m forest 30-I-2016 cow dung Pessoa MB. 1♀. [CEMT]. Goiânia GO080 4.5 km U[niversidade] F[ederal de] Goiás 16°35′1″S 49°15′4″W 763 m forest 31-II-2016 cow dung Pessoa MB. 1♂. [CEMT]. Leopoldo de Bulhões Serra Quebra Cangalha 16°41′39″S 48°50′34″W 903 m forest 6-III-2017 Pessoa MB. 2♂. [CEMT]. São Jorge Vale da Lua 14°10′52″S 47°47′24″W I-2011 ripfor-hum[an] faec[es] Nunes RV & Gigliotti MS. 2♂ 1♀. [CEMT]; same data but, [14°10′56″S 47°47′33″W]. 1♀. [CEMT]. Silvânia Fazenda Fiona 16°37′52″S 48°40′11″W 901 m forest 20-II-2017 human feces Pessoa MB. 1♂. [CEMT]. **Mato Grosso**″Araputanga Fazenda Bandeirantes 15°22′14″S 58°26′2″W 308 m Floresta semidecidual 20–22-I-2013 Pitfall hum-pig dung Silva RJ. 5♂ 3♀. [CEMT]; same data but, [15°23′6″S 58°26′2″W 19–21-I-2013]. 1♀. [CEMT]. Barra do Brugues Reserva Ecologica da Serra das Araras 27-I-1986 Marcolino S. 1♂. [CEMT]. Barra do Garças P[arque] E[stadual da] Serra Azul 15°50′26″S 52°14′46.2″W 516.3 m 12-XII-2012 Pitfall Silva JL. 1♂ 3♀. [CEMT]; same data but, [15°50′22.2″S 52°14′49″W 516.1 m 12-XII-2012]. 4♂ 1♀. [CEMT]; same data but, [15°50′27.5″S 52°14′46″W 515.9 m 12-XII-2012]. 1♂ 2♀. [CEMT]; same data but, [15°50′24.6″S 52°14′47″W 516.1 m 12-XII-2012]. 2♂ 1♀. [CEMT]; same data but, [15°50′5.9″S 52°14′21.3″W 675.8 m 06-XII-2012]. 1♀. [CEMT]; same data but, [15°51′6.03″S 52°14′49.5″W 08-XII-2012]. 1♀. [CEMT]; same data but, [15°50′17''S 52°13′29″W 738.8 m 08-XII-2012]. 1♀. [CEMT]; same data but, [15°51′5.3″S 52°14′48.5″W 08-XII-2012]. 1♀. [CEMT]. Barra do Garças Fazenda São Carlos 15°37′54″S 52°9′42″W 19-XI-2009. Aragona M. 3♂. [CEMT]. Chapada dos Guimarães 5-IV-2011 manual. 1♂. [CEMT]. Chapada dos Guimarães 05-XI-2016. 1♂. [CEMT]. Chapada dos Guimarães Cachoeira Geladeira 15°25′25″S 55°42′58″W 620 m 9-I-2013 pitfall Daniel GM. 1♀. [CEMT]. Chapada dos Guimarães Cachoeira Jamacá 15°27′40″S 55°43′2″W 771 m light Nunes RV, Cabra J. & Rossini M. 1♀. [CEMT]. Chapada dos Guimarães Jamacá 15°27′31″S 55°42′45″W 750 m 2–9-II-2013 FIT Daniel GM. 1♂ 2♀. [CEMT]; same data but, [15°27′50″S 55°42′45″W 760 m Mata úmida 9-I-2013 pitfall]. 1♀. [CEMT]; same data but, [15°27′51″S 55°42′47″W 790 m Mata úmida 9-I-2013 pitfall]. 2♀. [CEMT]; same data but, [15°27′52″S 55°42′45″W 750 m Mata úmida 9-I-2013 pitfall]. 3♂ 2♀. [CEMT]. Chapada dos Guimarães Trilha Casa do Mel 15°22′53″S 55°50′35″W 520 m XII-2015 ligth Nunes RV., Cabra J. & Rossini M. 1♂ 3♀. [CEMT]. Chapada dos Guimarães Vale do Jamacá 12-XII-2008 Pitfall/isca Freitas-Junior DS. & Moratelli A. 1♂. [CEMT]. Chapada dos Guimarães Vale do Jamacá 20-V-2015 Pitfall Santana E. et al. 4♂ 5♀. [CEMT]. Chapada dos Guimarães Veu de Noiva 15°24′21″S 55°50′10″W 540 m 25-I-2013 pitfall Daniel GM. 1♂ 2♀. [CEMT]; same data but, [15°24′23″S 55°50′10″W 510 m]. 1♂ 1♀. [CEMT]; same data but, [15°24′24″S 55°50′10″W 490 m]. 1♂. [CEMT]; same data but, [15°24′32″S 55°49′55″W 590 m Mata galería]. 1♀. [CEMT]. Cláudia 19-II-2013 Camera BF. 1♂. [CEMT]. Cláudia Fazenda Continental 11°25′33.4″S 55°18′7.9″W. 1♂. [CEMT]. Cláudia Fazenda Continental 11°36′55″S 55°15′21″W 30-VII-2010 Hum[an] dung. 1♀. [CEMT]. Cláudia Fazenda Continental 11°25′41″S 55°18′54″W 20-II-2011 Hum[an] dung Souza MF. 1♂ 1♀. [CEMT]; same data but, [11°35′39″S 55°17′7″W]. 1♂. [CEMT]; same data but, [11°36′29″S 55°15′1″W]. 2♂. [CEMT]. Cláudia PPBio 11°34′54″S 55°17′16″W 365 m 28-III-2010 mistnet Florêncio FP. 1♂. [CEMT]; same data but, [11°35′13″S 55°16′48″W 370 m]. 2♂ 3♀. [CEMT]; same data but, [11°36′35″S 55°15′48″W 390 m]. 1♂ 3♀. [CEMT]; same data but, [11°35′13″S 55°16′48″W 370 m]. 1♂ 1♀. [CEMT]; same data but, [11°35′32″S 55°16′21″W 370 m 27-III.−2012]. 1♀. [CEMT]; same data but, [11°35′50″S 55°15′55″W 365 m 27-III.−2012]. 3♀. [CEMT]; same data but, [11°36′10″S 55°15′28″W 350 m 25-III-2010]. 2♂ 1♀. [CEMT]; same data but, [11°36′29″S 55°15′2″W 360 m 24-III-2010]. 1♂ 1♀. [CEMT]; same data but, [11°35′20″S 55°17′35″W 360 m 22-III.−2012]. 2♀. [CEMT]; same data but, [11°35′39″S 55°17′7″W 380 m 22-III-2010]. 12♂ 18♀. [CEMT]; same data but, [11°35′57″S 55°16′41″W 380 m 21-III-2010]. 5♂ 10♀. [CEMT]; same data but, [11°24′57″S 55°19′2″W 335 m 31-III-2010]. 1♂. [CEMT]; same data but, [11°25′34″S 55°18′8″W 340 m 04-IV-2010]. 1♂. [CEMT]; same data but, [11°25′53″S 55°17′41″W 350 m 03-IV-2010]. 2♂ 1♀. [CEMT]; same data but, [11°25′53″S 55°17′41″W 350 m 04-IV-2010]. 1♂ 2♀. [CEMT]; same data but, [11°26′11″S 55°17′14″W 370 m 03-IV-2010]. 1♂ 3♀. [CEMT]; same data but, [11°25′42″S 55°18′54″W 340 m. 01-IV-2010]. 1♂. [CEMT]; same data but, [11°26′1″S 55°18′26″W 325 m 04-IV-2010]. 1♂ 2♀. [CEMT]; same data but, [11°38′51″S 55°5′21″W 370 m 12-III-2010]. 1♂ 1♀. [CEMT]; same data but, [11°38′44″S 55°4′36″W 365 m 14-III-2010]. 3♂ 3♀. [CEMT]; same data but, [11°38′25″S 55°5′2″W 365 m 15-III-2010]. 1♂ 2♀. [CEMT]; same data but, [11°37′46″S 55°5′56″W 370 m 17-III-2010]. 1♂. [CEMT]. Cocalinho Caverna Santa Terezinha AFL calcário 14º12′57″S 51º41′19″W FIT 15-XII-2016 Nunes R.V. 1♀. [CEMT]. Diamantino 13°48′07″S 56°41′32″W human feces 28-VI-2018 Nunes L.G. & E. Carvalho. 1♂. [CEMT]; same data but, [13°51′02″S 56°41′08″W]. 2♀. [CEMT]; same data but, [13°53′10″S 56°40′42″W]. 2♂ 2♀. [CEMT]; same data but, [13°48′51″S 56°41′32″W semidouro]. 4♀. [CEMT]; same data but, [13°49′14″S 56°40′51″W cerradinho 17-III-2018]. 1♂ 1♀. [CEMT]. Diamantino Alto do Rio Arinos II-2003 Furtado E. 1♂ 2♀. [CEMT]. Diamantino Reserva Vale da Solidão 14°22′S 56°07′W III-2013 Furtado E. 1♂. [CEMT]; same data but, [31-III-2020]. 1♂. [CEMT]. Diamantino Vale da Solidão 14°21′53″S 56°7′30″W mata ciliar 31-I-2009 I[nterceptação de] Voo Oliveira DCT. 1♂. [CEMT]; same data but, [14°22′13''S 56°7′12''W mata ripária 17-II-2009]. 1♂. [CEMT]. Diamantino Fazenda São João 14°17′5″S 56°16′14″W 17-XI-2013 pitfall with liver Maldaner ME. 1♀. [CEMT]. Figueiropolis d'Oeste Fazenda Monte Fusco 15°33′31″S 58°36′53″W 252 m Floresta semidecidual 11–13-I-2013 Pitfall hum-pig dung Silva RJ. 2♂. [CEMT]. Indiavai Fazenda Alto Jaurú 15°27′18″S 58°33′12″W 231 m Floresta semidecidual 14–16-I-2013 Pitfall hum-pig dung Silva RJ. 1♂ 1♀. [CEMT]; same data but, [15°26′19″S 58°34′51″W 308 m 12–14-I-2013]. 1♂ 1♀. [CEMT]; same data but, [15°26′31″S 58°35′39″W 252 m 14–16-I-2013]. 2♂ 1♀. [CEMT]. Marcelândia Fazenda Agromaster borda 10°51′37″S 54°47′59″W 350 m 15–17-XI-12018 Pitfall human dung Ferreira JV. 1♀. [CEMT]; same data but, [10°51′03″S 54°43′21″W 400 m]. 1♂. [CEMT]. Mirassol d’Oeste Fazenda Santa Helena 15°36′2″S 57°57′46″W 231 m Floresta semidecidual 24–26-I-2013 Pitfall hum-pig dung Silva RJ. 1♂. [CEMT]. Nova Mutum Trivelato XII-1995 Silva WO. 1♂ 2♀. [CEMT]. Nova Mutum Trivelato 15-XII-1995 Silva-filho WO. 1♂ 3♀. [CEMT]. Nova Mutum 13°48′7″S 56°5′22″W 23-I-2011 hum. Dung Souza MF. 1♀. [CEMT]; same data but, [25-I-2011]. 2♀. [CEMT]; same data but, [18-IV-2011]. 1♂ 1♀. [CEMT]; same data but, [02-V-2011]. 2♂ 2♀. [CEMT]. Nova Mutum Fazenda Mutum cerrado sensu stricto 13°56′19″S 55°57′12″W 20–22-XII-2018 pitfall human dung Machado AF. 1♀. [CEMT]; same data but, [13°47′21″S 56°00′45″W floresta]. 1♀. [CEMT]; same data but, [13°49′30″S 55°57′32″W floresta 19–21-XI-2018]. 1♀. [CEMT]. Nova Ubiratã ES[tação] EC[ológica] Ronuro 13°4′45″S 54°32′14″W 26-II-2017 pitfall hum[an] fec[es] Nunes LGOA. 6♂ 4♀. [CEMT]; same data but, [13°3′34″S 54°36′10″W]. 1♀. [CEMT]; same data but, [13°3′33″S 54°36′9″W]. 1♀. [CEMT]; same data but, [13°03′37″S 54°36′09″W]. 1♂ 1♀. [CEMT]; same data but, [13°04′36″S 54°32′35″W]. 3♂ 2♀. [CEMT]; same data but, [13°04′45″S 54°33′14″W]. 1♀. [CEMT]. Nova Ubiratã ES[tação] EC[ológica] Ronuro 13°06′36''S 54°26′38″W 27-II-2017 pitfall hum fec Nunes LGOA et al. 4♂ 2♀. [CEMT]. Nova Ubiratã ES[tação] EC[ológica] Ronuro 13°4′45″S 54°32′14″W 28-II-2017 pitfall hum fec Nunes LGOA. 1♂. [CEMT]. Nova Ubiratã ESEC Rio Ronuro 27-II-2017 Nunes LGOA. et al. 2♂. [CEMT]. Nova Ubiratã ES[tação] EC[ológica] Rio Ronuro 13°4′36″S 54°32′36″W 26-II-2017 dung Nunes LGOA. et al. 5♂ 1♀. [CEMT]. Novo Santo Antônio Vila Trindade 12°17′23″S 51°5′13″W 196 m Pitfall hum/pig dung Silva RJ.& Silva RSA. 2♂ 1♀. [CEMT]. Floresta alagada 18–20-II-2019 Pontes e Lacerda Mineracão Yamana P. Ernesto 15°20′36.4″S 59°15′9.3″W 01–06-III-2013 col. Manual e pitfall Ribeiro RAK. 1♂ 3♀. [CEMT]. Paranatinga 10–15-XI-2011 manual Neto O. 1♀. [CEMT]. Nova Ubiratã ES[tação] EC[ológica] Rio Ronuro 27-II-2017 dung Nunes LGOA. 3♂ 2♀. [CEMT]. Porto Estrela E[stação] E[cológica da] Serra das Araras 15°0′24″S 59°56′46″W 16-XI-2015 Chagas A. & Kury A. 2♀. [CEMT]. Porto Estrela E[stação] E[cológica da] Serra das Araras heliporto 15°39′19″S 57°12′50″W 22-XI-2017 Ribeiro RF. 1♀. [CEMT]. Porto Estrela E[stação] E[cológica da] Serra das Araras Trilha Boca do José 17–19-XI-2017 Pitfall Brum P. 16♂ 8♀. [CEMT]. Porto Estrela E[stação] E[cológica da] Serra das Araras Trilha Boca do José cerrado XI-2017 Pitfall Conceição TF. 2♀. [CEMT]; same data but, [15°38′19″S 57°12′13″W]. 1♀. [CEMT]; same data but, [15°38′28″S 57°12′18″W]. 1♂. [CEMT]; same data but, [semideciduous forest]. 2♀. [CEMT]; same data but, [galery forest]. 2♂ 2♀. [CEMT]. Porto Estrela E[stação] E[cológica da] Serra das Araras Trilha Boca do José XI-2017 Machado RJP. 2♀. [CEMT]. Porto Estrela ES[tação] EC[ológica da] Serra das Araras mata ciliar 15-X-2011 Pitfall Vaz-de-Mello FZ. 4♂ 6♀. [CEMT]; same data but, [15°38′59″S 57°12′38″W]. 1♂. [CEMT]. Porto Estrela ES[tação] EC[ológica da] Serra das Araras Trilha Boca do José 15°21′S 56°57 W cerrado 12-X-2011 Vaz-de-Mello FZ. 2♀. [CEMT]; same data but, [15°39′S 57°12.7′W 222 m cerradão 15-X-2011 Pitfall]. 1♂. [CEMT]; same data but, [15°38′59″S 57°12′38″W dry for[est] 188 m 10-X-2011 pitfall]. 1♀. [CEMT]. Porto Estrela Est[ação] Eco[lógica da]. Serra das Araras 15°39′10″S 57°12′30″W X-2013 Nunes RV. 1♂ 1♀. [CEMT]. Querência Fazenda São Luiz 12°39′930″S 52°21′685″W floresta amazónica II-2009 Pitfall com fezes humanas Andrade R. 1♂. [CEMT]; same data but, [12°39′728″S 52°21′742''W]. 1♂. [CEMT]; same data but, [12°39′288″S 52°21′775″W]. 1♀. [CEMT]. Querência Fazenda Tanguro forest − 12.99281S − 52.34204W human feces 20-III-2018 Ribeiro VS. 1♀. [CEMT]; same data but, [− 12.99320S − 52.34097W]. 1♂ 2♀. [CEMT]; same data but, [− 12.99388S − 52.34000W]. 3♂ 1♀. [CEMT]; same data but, [− 12.99464S − 52.33902W]. 2♂ 1♀. [CEMT]; same data but, [− 12.94888S − 52.42958W 21-III-2018]. 1♂ 3♀. [CEMT]; same data but, [− 12.94958S − 52.43044W 21-III-2018]. 1♂. [CEMT]; same data but, [− 12.94988S − 52.43331W 21-III-2018]. 1♂. [CEMT]; same data but, [− 12.87201S − 52.41082W 27-III-2018]. 1♂. [CEMT]; same data but, [− 12.87273S − 52.41020W 27-III-2018]. 1♀. [CEMT]; same data but, [− 12.99145S − 52.44435W 27-III-2018]. 1♂ 2♀. [CEMT]; same data but, [− 12.99210S − 52.44347W 27-III-2018]. 1♂. [CEMT]; same data but, [− 12.99288S − 52.44157W 27-III-2018]. 3♂ 1♀. [CEMT]; same data but, [−12.99327S −52.44054W 27-III-2018]. 3♂ 1♀. [CEMT]; same data but, [− 12.82960S − 52.44742W 03-IV-2018]. 5♂ 1♀. [CEMT]; same data but, [− 12.83010S − 52.44742W 03-IV-2018]. 1♂ 3♀. [CEMT]; same data but, [− 12.83046S − 52.44517W 03-IV-2018]. 2♀. [CEMT]; same data but, [− 12.88111S − 52.36240W 03-IV-2018]. 1♂. [CEMT]; same data but, [− 12.83471S −52.3329W 04-IV-2018]. 1♂ 1♀. [CEMT]; same data but, [−12.83539S −52.33503W 04-IV-2018]. 4♂ 3♀. [CEMT]; same data but, [−12.83566S −52.33963W 04-IV-2018]. 1♀. [CEMT]; same data but, [− 12.88066S − 52.36016W 04-IV-2018]. 2♂. [CEMT]; same data but, [− 12.88074S − 52.36135W 04-IV-2018]. 1♂. [CEMT]; same data but, [− 12.88111S − 52.36240W 04-IV-2018]. 2♂ 1♀. [CEMT]; same data but, [− 12.88157S − 52.36330W 04-IV-2018]. 1♂. [CEMT]; same data but, [− 12.88215S − 52.36431W 04-IV-2018]. 1♀. [CEMT]; same data but, [− 12.72903S − 52.38032W 10-IV-2018]. 1♀. [CEMT]; same data but, [− 12.72907S − 52.33274W 10-IV-2018]. 1♂. [CEMT]; same data but, [− 12.72961S − 52.32920W 10-IV-2018]. 1♂. [CEMT]; same data but, [− 12.73017S − 52.32820W 10-IV-2018]. 3♂. [CEMT]; same data but, [− 13.09675S − 52.35938W 10-IV-2018]. 1♂. [CEMT]; same data but, [− 13.09675S − 52.36078W 10-IV-2018]. 1♀. [CEMT]; same data but, [− 13.09708S − 52.36224W 10-IV-2018]. 1♂ 1♀. [CEMT]; same data but, [− 13.09787S − 52.36325W 10-IV-2018]. 1♂. [CEMT]; same data but, [− 12.97000S − 52.39771W 20-IV-2018]. 2♀. [CEMT]; same data but, [− 12.97000S − 52.39771W 20-IV-2018]. 1♂ 4♀. [CEMT]; same data but, [− 12.97779S − 52.39559W 20-IV-2018]. 7♂ 9♀. [CEMT]; same data but, [− 12.97801S − 52.39455W 20-IV-2018]. 1♂ 1♀. [CEMT]; same data but, [− 12.97833S − 52.39369W 20-IV-2018]. 1♂ 2♀. [CEMT]. Querência Fazenda São Luis 12°38.89′S 52°22.5′W estaçao chuvosa II-2009 FIT Andrade R. 1♂. [CEMT]; same data but, [12°39.32′S 52°21.93′W]. 1♂. [CEMT]; same data but, [12°38.9′S 52°22.83′W pitfall]. 1♂. [CEMT]; same data but, [12°39.95′S 52°21.93′W pitfall]. 1♀. [CEMT]. Rosário Oeste SESC Serra Azul 14°29′57″S 55°44′02″W Ligth trap 18–19-IV-2023 Rodrigues D.F. et al. 1♀. [CEMT]. Santo Antônio do Leverger São Vicente da Serra 15°49′42″S 55°25′11″W mata 19–21-I-2011 hum dung Tissiani ASO. 1♂. [CEMT]; same data but, [15–17-XII-2010]. 3♂. [CEMT]. Santo Antônio do Leverger São Vicente da Serra Campus I[nstituto] F[ederal de Educação Ciência e Tecnologia de] M[a]T[o] Grosso 15°49′42″S 55°25′11″W forest 08–10-X-2011 hum dung Tissiani & Vaz-de-Mello. 2♂ 4♀. [CEMT]. [Santo Antônio do Leverger] São Vicente da Serra I[nstituto] F[ederal de Educação Ciência e Tecnologia de] M[a]T[o] Grosso 15°49′36″S 55°24′47″W V-2011 Neves GAPC. 1♂. [CEMT]. São Félix do Araguaia Fazenda Trinta Lagos 11°35′52″S 50°42′15″W 181 m Mata de galeria 23-II-2019 Pitfall hum/pig dung Silva R.J. & Silva R.S.A. 1♂ 1♀. [CEMT]. São José de Rio Claro 13°53′10″S 56°40′42″W human feces 16-III-2018 Nunes L.G. & Carvalho E. 1♂. [CEMT]; same data but, [30-VI-2018]. 11♂ 9♀. [CEMT]. Sorriso Fazenda San Antônio Cerradão 12°54′54″S 55°49′11''W 393 m Pitfall human dung 16-XII-2018 Machado AF. 2♂. [CEMT]; same data but, [12°55′08″S 55°44′02''W 414 m 18-XII-2018]. 1♂. [CEMT]; same data but, [Cerrado sensu stricto 12º53′35″S 55°52′32″W 396 m 19-XII-2018]. 1♂. [CEMT]. Tangará da Serra Fazenda Ap. da Serra 14°19′15″S 57°43′51″W 640 m cerrado 20–22-IV-2017 pitfall hum dung Silva RJ. 1♀. [CEMT]. Tangará da Serra Fazenda Bahía 14°37′19″S 57°25′7″W 419 m Floresta semidecidual 12–14-I-2011 Pitfall hum-pig dung Silva RJ. 5♂ 3♀. [CEMT]; same data but, [14°37′13″S 57°24′50″W]. 1♂ 1♀. [CEMT]; same data but, [14°37′13″S 57°24′54″W]. 1♀. [CEMT]. Tangará da Serra Fazenda Filé do Boi 14°38′7″S 57°24′41″W 439 m Floresta semidecidual 25–27-I-2011 Pitfall cow dung Silva RJ. 1♂. [CEMT]. Tangará da Serra Fazenda Fontosa 14°35′47″S 57°50′40″W 322 m floresta semidecidual 06-II-2012 Pitfall hum-pig dung Silva RJ. 4♂. [CEMT]; same data but, [14°36′2″S 57°50′15″W 310 m 31-I-2012]. 1♂. [CEMT]; same data but, [14°36′4″S 57°50′28″W 312 m 06-II-2012]. 1♂. [CEMT]; same data but, [14°36′2″S 57°50′19''W 313 m 31-I-2012 Pitfall cow dung]. 1♂ 1♀. [CEMT]; same data but, [14°35′49″S 57°50′37″W 318 m 07–14-II-2012 FIT]. 1♀. [CEMT]. Tangará da Serra Fazenda Netolandia 14°39′54″S 57°55′8″W 329 m Floresta semidecidual 12–14-III-2012 Pitfall hum-pig dung Silva RJ. 2♂ 5♀. [CEMT]; same data but, [14°41′5″S 57°54′8″W 263 m 25–27-III-2012]. 1♂. [CEMT]; same data but, [14°42′18″S 57°54′14″W 310 m 12–14-XII-2012 Pitfall cow dung]. 1♀. [CEMT]; same data but, [14°42′18″S 57°54′14″W 310 m 14–21-XII-2012 FIT]. 1♂. [CEMT]. Tangará da Serra Fazenda Rosa Branca 14°34′0″S 57°52′24″W 468 m Floresta semidecidual 25–27-II-2011 Pitfall hum-pig dung Silva RJ. 3♂. [CEMT]. Tangará da Serra Fazenda Sudamata 14°37′32″S 57°58′12″W 336 m Floresta semidecidual 15–17-II-2012 Pitfall hum-pig dung Silva RJ. 8♂ 6♀. [CEMT]; same data but, [14°37′18″S 57°58′1″W 354 m]. 2♂. [CEMT]; same data but, [14°37′18″S 57°58′1″W 354 m 21–23-II-2012]. 1♀. [CEMT]; same data but, [14°37′18″S 57°58′1″W 354 m]. 2♂. [CEMT]; same data but, [14°38′13″S 57°56′8″W 310 m 20–22-II-2012]. 1♂. [CEMT]; same data but, [14°37′19″S 57°58′4″W 332 m 15–17-II-2012]. 1♀. [CEMT]; same data but, [14°37′19″S 57°58′4″W 332 m 10–17-III-2012 FIT]. 1♀. [CEMT]; same data but, [14°38′36″S 57°57′44″W 346 m 10–17-III-2012 FIT]. 1♀. [CEMT]. Tangará da Serra Sitio Clemente 14°39′46″S 57°49′35″W 368 m Floresta semidecidual 12–14-XII-2012 Pitfall hum-pig dung Silva RJ. 2♂. [CEMT]. Tangará da Serra Fazenda Promissão Floresta semidecidual 14°30′2″S 57°17′28″W Pitfall human dung 24–26-VII-2017 Santos UF. 3♂. [CEMT]. Vera Floresta 12°23′05″S 55°15′12″W FIT 05–12-XII-2018 Machado AF. 1♂ 1♀. [CEMT]; same data but, [Fazenda Pedro 12°19′11″S 55°21′34″W pitfall human dung]. 1♀. [CEMT]. **Mato Grosso do Sul**: Aquidauana 20°26′54″S 49°38′39″W fragmento do Cerrado 15-X-2011 armadilha pitfall (fezes suinas) Correa CMA. 2♂. [CEMT]. Bodoquena Fazenda California 20°41′8″S 56°51′31″W III-2011 hum. faec Bavutti LO. 6♂ 3♀. [CEMT]. Corumbá Fazenda São Bento 17°19′19″S 56°41′51″W Floresta ciliare Rio Piquiri 28-IV-2012 Rossini M. 1♂ 2♀. [CEMT]; same data but, [17°19′14″S 56°41′55″W]. 1♀. [CEMT]; same data but, [17°19′16″S 56°42′5″W]. 1♀. [CEMT]; same data but, [17°19′15″S 56°42′7″W]. 1♀. [CEMT]; same data but, [17°19′18″S 56°42′20″W]. 1♀. [CEMT]. Dourados 17-VIII-2008 Rodrigues MM. 1♂ 1♀. [CEMT]. Dourados 16-XII-2006 Miloca M. 1♀. [CEMT]. Ivinhema 22°31′40″S 53°53′38″W III-2011 hum. Faec. Bavutti LO. 1♀. [CEMT]. Selviria UN[niversidade] ES[tadual] P[aulista] farm riparian forest-pasture transition área 19-II-1993 carrion baited pitfall trap Flechtmann CAH. 1♂ 1♀. [CEMT]; same data but, [12-I-1993]. 1♀. [CEMT]; same data but, [16-II-1993]. 1♀. [CEMT]; same data but, [*Brachiaria decumbens* pasture 3-II-2000 black ligth trap]. 1♀. [CEMT]. Selviria UN[niversidade] ES[tadual] P[aulista] farm Atlantic forest fragment 20°22′58.37″S 51°24′38.09″W bovine dropping baited pitfall 20-II-2007 Mesquita F.W. 1♂ 1♀. [CEMT]. Selviria UN[niversidade] ES[tadual] P[aulista] farm Cerradão fragment 20°20′12.7″S 51°24′32.4″W *Bos taurus* dropping baited pitfall trap 25-X-2007 Oikawa F. 2♂ 1♀. [CEMT]; same data but, [31-X-2007]. 1♀. [CEMT]; same data but, [12-XII-2007]. 1♀. [CEMT]; same data but, [24-IV-2008]. 1♂. [CEMT]; same data but, [31-X-2007 human feces baited pitfall trap]. 1♂. [CEMT]; same data but, [25-X-2007 human feces baited pitfall trap]. 1♀. [CEMT]; same data but, [14-XI-2007 human feces baited pitfall trap]. 1♂. [CEMT]; same data but, [11-XI-2007 *Sus scrofa* feces baited pitfall trap]. 1♂. [CEMT]; same data but, [07-XI-2007 *Sus scrofa* feces baited pitfall trap]. 1♂. [CEMT]; same data but, [08-XI-2007 *Sus scrofa* feces baited pitfall trap]. 2♀. [CEMT]; same data but, [28-XI-2007 *Sus scrofa* feces baited pitfall trap]. 1♀. [CEMT]; same data but, [27-II-2008 *Sus scrofa* feces baited pitfall trap]. 1♀. [CEMT]; same data but, [28-II-2008 *Sus scrofa* feces baited pitfall trap]. 1♂. [CEMT]; same data but, [09-IV-2008 *Sus scrofa* feces baited pitfall trap]. 1♀. [CEMT]; same data but, [17-IV-2008 *Sus scrofa* feces baited pitfall trap]. 1♂. [CEMT]; same data but, [31-X-2007 *Tayassu tajacu* feces baited pitfall trap]. 1♀. [CEMT]; same data but, [24-IV-2007 *Tayassu tajacu* feces baited pitfall trap]. 1♀. [CEMT]. Três Lagoas Fazenda Barra do Moeda XI-2009 Rosas PFC. 1♀. [CEMT]. Três Lagoas Horto Barra do Moeda, Três Lagoas Agroforestal Cerrado-*Eucalyptus grandis* ecotone black ligth trap 23-XI-1993 Flechtmann C.A.H. 1♂. [CEMT]; same data but, [Cerrado standard 02-XI-1993]. 1♀. [CEMT]; same data but, [Cerrado standard 30-III-1993 ethanol-baited FIT]. 1♀. [CEMT]. **Minas Gerais**: Brumadinho X-1946 Penna F. 1♂. [CEMT]. Conceição dos Ouros Rio Sapucaí 22-XI-2004 Almeida-Neto G.P. & Pereira E.A. 1♂. [CEMT]. Cordisburgo Fazenda Rontinha XII-1998 Vaz-de-Mello. 1♂. [CEMT]. Curvelo Fazenda do Roberto 18°47′43″S 44°38′51″W 655 m 26-I-2012 human dung Macedo R. 2♀. [CEMT]. Ibitira XI-1988 luz. 1♀. [CEMT]. Ituiutaba 15-VIII-1999 Vilarinho ES. 1♂. [CEMT]. Paracatu XII-1997 Lourenço Jr S. 1♂ 2♀. [CEMT]. Paraopeba XI-1996 Lourenço Jr S. 2♂ 3♀. [CEMT]. Santana do Riacho PAR[que] NA[cional da] Serra do Cipó mata 11-XI-2004 Schiffler G. 1♂. [CEMT]. Tabocas XI-1990 luz. 2♂. [CEMT]. Ipaba 19°20′16″S 42°25′2″W II-2020 pitfall (human feces) Macedo-Reis LE. 1♀. [CEMT]. **São Paulo**″Agudos Duaflora S.A. *Pinus caribaea* v. *hondurensis* Stand 16-XI-1993 Ten trap w P. car v. car logs Flechtmann CAH. 1♂. [CEMT]; same data but, [26-I-1993]. 1♂ 1♀. [CEMT]; same data but, [02-III-1993]. 1♀. [CEMT]; same data but, [30-III-1993]. 1♂. [CEMT]; same data but, [control ten trap]. 1♀. [CEMT]; same data but, [11-I-1994 ethanol-baiterd fligth interception trap]. 1♂. [CEMT]; same data but, [01-II-1994 ethanol-baiterd fligth interception trap]. 1♂. [CEMT]. Cajuru rural area hand collected 03-I-2016 Longo L. 1♀. [CEMT]. Itirapina Estação Ecológica 22°14′28.3″S 47°49′56.8″W Cerrado 30-I-2009 Silva F. 2♂ 2♀. [CEMT]. Ribeirão Preto E[stação] E[cológica] R[ibeirão] P[reto] Mata Santa Tereza 6-I-2006 luz Polegatto & Nascimento. 1♀. [CEMT]; same data but, [malaise]. 1♀. [CEMT]. Teodoro Sampaio Morro do Diabo State Reservation 26-V-1993 bovine dropping baited pitfall trap Teixeira LL. 1♂. [CEMT]; same data but, [10-II-1993]. 1♀. [CEMT]; same data but, [9-IV-1994]. 1♀. [CEMT]; same data but, [15-XII-1993]. 1♀. [CEMT]; same data but, [17-II-1993 bugio dung baited pitfall trap]. 1♂. [CEMT]. **Tocantins**: Pium Fazenda Quero Quero 10-X-2016 pitfall Medeiros LA. 5♂ 5♀. [CEMT]. Porto Nacional Perto a Fazenda Giovam 10°5.87'S 48°27.43'W 245 m 02-XII-2013 Chagas & Giupponi. 1♀. [CEMT]. Porto Nacional Fazenda Supapo-Zé Ferreira 10°5′40″S 48°25′51″W 245 m 30-XII-2013 Chagas & Giupponi. 1♀. [CEMT]. All Paratypes have a second label: 2: {printed text on yellow label with black margins} *Dichotomius barberoi* sp. nov. Arias-Buriticá & Vaz-de-Mello, 2026 [♂ or ♀] **PARATYPE**.

#### Diagnosis

This species can be distinguished from the other members of the *D*. *depressicollis* species group by the following combination of characters: both sexes with pronotal process with the axes formed between their tips forming a cross (†) (Fig. 4), elytra with striae distinctly impressed, shagreened, and regularly interrupted by shallow punctures (Fig. 5b), metaventrite near to the insertion of metacoxa without setae (Fig. 5a), metatrocanther without setae (Fig. 5a), morphology of the male genital organ as in Fig. 5c–g, and shape of endophallites Fig. 5h–k).

#### Etymology

This species is named in memory of the Italian scarabaeoidologist Enrico Barbero (1954–2025), a good friend of FZVM, who in his youth encouraged his interest in the study of *Dichotomius* taxonomy.

#### Description

**Male**: Length 23.5 mm; width 14.2 mm (Fig. 4a). On the top of the pronotum in the central area, which reaches to the medial area, it has a central process forming a cross (†) with its axes, with three pronounced points and the central one with one lobe with triangular appearance (Fig. 4a–c). Surface of the pronotal disk with large and deep punctures presenting microsculpture inside (seen at high magnification) separated less than their diameter, dense in the posterocentral region of the pronotal disk and decreasing towards the apex of the pronotal protuberances (Fig. 4b). **Metaventrite**: Postero-central region of metaventrite near to insertion of metacoxa without setae (Fig. 5a). **Elytra**: Striae bicarinate, with striae distinctly impressed, shagreened, and regularly interrupted by shallow punctures (Fig. 5b). Interstriae with shiny surface, with distinct wrinkles in basal area and less distinct in apical area, with small and shallow punctures, separated by three times their diameter and visible under low magnification (Fig. 5b). **Abdomen**: third to fifth ventrites without lateral setae (Fig. 5l).

#### Aedeagus

Parameres are triangular in lateral view (Fig. 5c). In dorsal view, parameres are symmetrical; with a widened base, decreasing in thickness towards the apical region ending in a rounded tip with short setae of equal length in all extensions (Fig. 5f). Left paramere with a subgenital plate partially visible (Fig. 5d). In ventral view, parameres are broad at the basal region with an acute prolongation; the apical region is narrow with a rounded apex without setae (Fig. 5g). A subgenital plate is present, broad, protruding beyond the paramere on the right side (Fig. 5e). **Endophallites**: ME is large and subquadrangular with two well sclerotized processes on the right lateral side, the surface covered with bristles along its entire length, and on the left side, it presents a membranous process with long bristles and with the spine toward the right edge (Fig. 5k). A+SA complex is sclerotized in the central area surrounded by a semi-sclerotized membrane (Fig. 5i). SRP has a “C” shape with an acute and sclerotized projection at the base (Fig. 5h). FLP has an “N” shape (Fig. 5j).

#### Variation

In males, length varies from 18.2 to 26.6 mm and the width from 11.2 to 16.3 mm; the cephalic process varies in length from 0.2 to 1.1 mm. **Females**: the length varies from 19.6 to 24.4 mm and the width from 12.0 to 15.8 mm; the cephalic process varies in length from 0.2 to 0.9 mm. In the pronotum, the central process is rounded at the anterior margin (Fig. 4d–f). Sixth abdominal ventrite is not compressed toward the middle region.

#### Distribution and Ecology

Brazil in the states of Goiás, Mato Grosso, Mato Grosso do Sul, Minas Gerais, São Paulo, Tocantins, and Distrito Federal (Fig. 3). Captured mainly in the Brazilian Cerrado, but sometimes captured in gallery forest and humid forest. It was collected from October to July, between 188 and 903 m.a.s.l., using pitfall traps baited with human, cow, pig or *Tayassu tajacu* (collared peccary) dung, carcasses, liver; manual collection, mist net, light trap and flight interception trap (FIT).

3. ***Dichotomius (Dichotomius) davidedmondsi*** **Arias-Buriticá & Vaz-de-Mello sp. nov**

http://zoobank.org/urn:lsid:zoobank.org:act:DB209C06-5AE2-499E-B7F5-54A8F5729445

(Figs. 6a–f, 7a–l, 3 starts)

#### Type Material

Holotype**: 1**♂. Labels: 1: {printed text on white label}: **BOLIVIA**: **Santa Cruz**: Provincia Chiquitos 1.6 km ENE Santiago 18°20.103′S 59°35.007'W 622 m Chiquitano dry forest XI-2008 human feces Edmonds WD. & Vidaurre T./2: {printed and handwritten text on red label} **HOLOTYPE** *Dichotomius davidedmondsi* sp. nov. Arias-Buriticá & Vaz-de-Mello, 2026. [CEMT]./3: {printed text on white label with black margins} QR code CEMT CUIABÁ 00114775. **Paratypes**: same data of holotype. 23♂ 34♀. [CEMT]. **BOLIVIA**: **La Paz**: Provincia Nor Yungas Cotapata 19-IX-2000 Y. Gareca. 1♂. [MHNNKM]. **Santa Cruz**: Buena Vista 1700 ft II-IV-1925 Collection E.R. Leach. 1♀. [CEMT]. Noel Kempff Mercado National Park 1997 S. Spector. 1♀. [CALT-ECC]. Provincia Chiquitos San José [de Chiquitos] 16-I-2010 T. Vidaurre. 2♀. [MHNNKM]. Provinca Chiquitos Serranía de Santiago de Chiquitos 18°12'S; 59°35'W 150 m 09-IV-2005 C. Hamel & T. Vidaurre. 2♂ 2♀. [MHNNKM]; same data but, 11-IV-2005. 1♂. [MHNNKM]. Provincia Cordillera [Cabezas] R[eserva] M[unicipal] Parabanó, Corralones k0447034; 7943514, 19–30-V.2004 T. Vidaurre. 5♂ 4♀. [MHNNKM]. Provincia Cordillera S[erra]nía Parabanó 13–14-III-2002 L. Cespedes. 1♂. [MHNNKM]. All Paratypes have a second label: 2: {printed text on yellow label with black margins} *Dichotomius davidedmondsi* sp. nov. Arias-Buriticá & Vaz-de-Mello, 2026 [♂ or ♀] **PARATYPE**.

#### Diagnosis

This species can be distinguished from the other members of the *D*. *depressicollis* species group by the following combination of characters: both sexes with pronotal process with the axes formed between their tips forming a cross (†) (Fig. 5b, e), elytra with striae distinctly impressed, shagreened, and regularly interrupted by shallow punctures (Fig. 7b), metaventrite near the insertion of the metacoxa with some short setae (Fig. 7a), metatrocanther without setae (Fig. 7a), metaventrites I–V with lateral setae (Fig. 7l), morphology of the male genital organ as in Fig. 7c-g, and shape of endophallites (Fig. 7h-k).

#### Etymology

This species is named in honor of Dr. David Edmonds in recognition of his contributions to the taxonomy, ecology, and conservation of dung beetles, and he collected the type series of this species.

#### Description

**Male**: Length 21.3 mm; width 12.8 mm (Fig. 6a). On the top of the pronotum in the central area, which reaches to the medial area, it has a central process forming a cross (†) with its axes, with three pronounced points and the central one with one lobe with a triangular appearance (Fig. 6a–c). Surface of the pronotal disk with large and deep punctures presenting microsculpture inside (seen at high magnification) separated less than their diameter, dense in the posterocentral region of the pronotal disk and decreasing toward the apex of the pronotal protuberances where they are separated twice their diameter. **Metaventrite**: Postero-central region of the metaventrite near to the insertion of the metacoxa without setae (Fig. 7a). **Elytra**: Striae bicarinate, with striae distinctly impressed, shagreened, and regularly interrupted by shallow punctures (Fig. 7b). Surface of interstriae with a wavy appearance in all extensions, with deep and distinct punctures, separated by three times their diameter and visible under low magnification (Fig. 7b). **Abdomen**: third to fifth ventrites with lateral setae (Fig. 7l).

#### Aedeagus

Parameres triangular with acute apex in lateral view (Fig. 7c). In dorsal view, parameres asymmetrical, the right one being shorter; with a widened base, decreasing in thickness towards apical region ending in a rounded tip with short setae of equal length in all extensions (Fig. 7f). Left paramere with subgenital plate partially visible (Fig. 7d). In ventral view, parameres broad at basal region with an acute prolongation; the apical region is narrow with rounded apex with short setae (Fig. 7g). A subgenital plate is present, broad, protruding beyond the paramere on the right side (Fig. 7e). **Endophallites**: ME large and subquadrangular with two well sclerotized processes on the right lateral side, the surface covered with bristles along its entire length, and on the left side it presents a membranous process with long bristles and with the spine toward the superior right edge (Fig. 7k). As+SA complex is sclerotized in the central area surrounded by a semi-sclerotized membrane (Fig. 7i). SRP has a “C” shape, wide, with a wide base sclerotized (Fig. 7h). FLP has an “N” shape (Fig. 7j).

#### Variation

In males, length varies from 15.7 to 25.6 mm and the width from 10.3 to 15.5 mm; the cephalic process varies in length from 0.3 to 1.1 mm. **Females**: the length varies from 17.6 to 25.3 mm and the width from 11.2 to 16.6 mm; the cephalic process varies in length from 0.2 to 0.9 mm. In the pronotum, the central process is rounded at the anterior margin (Fig. 6d–f). Sixth abdominal ventrite is not compressed toward the middle region.

#### Distribution and Ecology

Bolivia in the La Paz and Santa Cruz department (Fig. 3). It was collected in the Chiquitano dry forest in January, March, April, and November, from 518 to 622 m.a.s.l., using pitfall traps baited with human dung.

4. ***Dichotomius (Dichotomius) depressicollis*** **(Harold, **1867**)**

(Figs. 8a–f, 9a–k, 3 circles)

*Pinotus depressicollis* Harold 1867: 98 (Original description)

#### Material Examined

**Lectotype** [♂, here designated]: Labels: 1: {handwritten text on white label with red margins} Brasilia [Brazil] *P. depressicollis* t. Harold. [MNHN]. **Paralectotype** [1♂ and 1♀]: Labels: 1: {handwritten text on white label with red margins} Brasilia [Brazil] *P. depressicollis* t. Harold. [MNHN].

In the original description of *Pinotus depresicollis*, Harold (1867) mentions characteristics of males and females, but does not mention the number of specimens. He mentions the type locality, Brasilia, [now Brazil]. According to Horn et al. (1990), Harold’s collection was deposited at the Muséum National d’Histoire Naturelle (MHNM, Paris, France) via M. Korb and R. Oberthür in 1952. In this collection, FZVM found two males and one female, and we here designate the larger male specimen as the lectotype, the others as paralectotypes.

#### Non Type Specimens

**ARGENTINA: Misiones**: Andresito Península 10-I-2013 Zurita GA & Capello V. 1♀. [CEMT]. Puerto Libertad APSA [Alto Paraná S.A.] Península I-2010 Coprotrampa Payras MN. 1♂. [CEMT]. Puerto Libertad APSA [Alto Paraná S.A.] 25°48′1.48″S 54°23′0.54″W 18-I-2013 Zurita GA, Gómez A. & Giménez V. 1♀. [CEMT]. Puerto Libertad APSA [Alto Paraná S.A.] Límite R[eserva] P[rotectora] San Jorge 25°48′1.48″S 54°23′0.54″W 4-V-2002 Capello MV. 1♀. [CEMT]. **BRAZIL**: **Espírito Santo**: Domingos Martins P[ar]q[ue] E[stadual] Pedra Azul I-2000 Lopes-Andrade & Vaz-de-Mello. 1♂ 1♀. [CEMT]. Jacaraípe VI-1989. 1♂. [CEMT]. Santa Teresa Estação Biológica de Santa Lúcia Trilha Indaia-Açu 19°58′1″S 40°32′18″W 679 m 30-I-2015 pitfall human feces Vargas T. 1♀. [CEMT]. Vargem Alta 15-IX-1995 Louzada & Louzada. 2♂. [CEMT]; same data but, [IX-1995 Pitfall Louzada JNC]. 2♂. [CEMT]. Venda Nova do Imigrante Vila Santa Cruz 20°20′2″S 41°8′18″W 800 m 10–14-I-2011 hum[an] dung Vaz-de-Mello FZ. 1♀. [CEMT]. Venda Nova do Imigrante Lavrinhas 20°18′16″S 41°6′58″W I-2013 pitfal f[eces] Hum[anas] Vaz-de-Mello LF. 2♂. [CEMT]. **Mato Grosso do Sul**: Três Lagoas Horto Barra do Moeda Cerrado Standart 02-III-1993 Black light trap Flechtmann C.A.H. 1♀. [CEMT]. Selviria UNESP's Farm riparian forest area cattle dropping baited pitfall trap 27-II-1993 Rodrigues S.R. 1♀. [CEMT]. **Minas Gerais**: 1400 m XII-1998 Vaz-de-Mello FZ. 1♂ 1♀. [CEMT]. Diamantina Campus U[niversidade] F[ederal dos] V[ales do] J[equitinhonha e] M[ucuri] 19-XII-2005 Assis Junior SL. 9♂ 8♀. [CEMT]. Ipatinga I-1998 Grossi E. & Grossi P. 1♀. [CEMT]. Lavras V-1997 Louzada J. 2♀. [CEMT]. Lavras Mata do Capivari 21°16′25″S 44°16′57″W XII-2001 Schiffler G. 1♂ 1♀. [CEMT]. Nova Lima P[arque] E[stadual] Serra Rola Moça 2005 Schiffler G. 3♂ 2♀. [CEMT]. Paraopeba XI-1996 Lourenço Jr. S. 1♂. [CEMT]. Prados APA 21°4′40″S 44°8′6.1″W 1090 m 17-II-2012 pitfall com fezes humanas Vieira Leticia et al. 1♀. [CEMT]. Rio Acima Vale Mineração 20°3′27″S 43°40′23″W 1334 m forest 10-X-2010 human dung Mota RN. 1♀. [CEMT]. Santana do Riacho Serra do Cipó 19°15′11″S 43°33′7″W II-2015 Ferreira LF. 1♂. [CEMT]. Viçosa Mato do Paraiso XI-1987 ligth Fiúza PS. 1♀. [CEMT]. Viçosa 22-I-1992 Lopes & Louzada. 1♂. [CEMT]; same data but, [XII-1992 Vaz-de-Mello]. 1♂. [CEMT]; same data but, [2-I-1994 Louzada JNC]. 1♂. [CEMT]; same data but, [4-I-1994 Louzada JNC]. 1♂ 2♀. [CEMT]; same data but, [X-1994 Vaz-de-Mello]. 1♂. [CEMT]; same data but, [3-II-1994 Louzada JNC]. 1♀. [CEMT]; same data but, [pastagens 1994–1995 Louzada, Carvalho & Silva]. 1♀. [CEMT]. **Paraná**: Morretes Santuário Nhundiaquara 25°25′32″S 48°53′21″W 100 m 5-II-2016 pig feces Raine EH.1♀. [CEMT]. P[arque] N[atural] Foz do Iguaçu 25-II-1989 Robson JR & Bertolossi F. 1♂ 1♀. [CEMT]. Telêmaco Borda Klabin Papel e Celulose *Pinus taeda* stand. Ethanol-baited multiple funnel trap 05-IV-2002 Flecthmann C.A.H. 1♂. [CEMT]. **Rio de Janeiro**: P[arque] N[acional do] Itatiaia I-1993. 1♂. [CEMT]. Nova Friburgo XI-1993 Grossi E. 2♂. [CEMT]; same data but, [I-1995 Vaz-de-Mello FZ]. 2♂. [CEMT]. Pedro do Rio II-2011 Bello A. 1♂. [CEMT]. Petrópolis XII-1986. 1♀. [CEMT]. Petrópolis II-1992 Cordeiro N. 2♂. [CEMT]. **Rio Grande do Sul**: Derrubadas P[arque] E[stadual do] Turvo 27°8′44″S 53°53′10″W 07-I-2017 Armadilha queda Cassenote S. 1♂ 1♀. [CEMT]. **Santa Catarina**: Campos Novos 27°23′S 51°12′W II-2014 Pitfall isca Campos RC. 1♀. [CEMT]. Monte Castelo 26°40′S 50°18′W pinus 1987 837 m 24-XII-2012 pitfall Brandl AL. 1♀. [CEMT]. **São Paulo**: Bragança Paulista 22°57′30.2″S 46°37′37.97″W 872 m 12-III-2016 Fezes suínas + humana Tavares A. 1♂. [CEMT]. Campos do Jordão h. Florestal 20-XII-1998 Coleção GP Almeida isca fezes humanas Almeida Lincoln. 1♂. [CEMT]. Campos do Jordão 10-I-1999 Pereira EA. 1♂. [CEMT]. Mogi das Cruzes Parque das Neblinas 23°47′57″S 46°13′7″W 25-II-2015 mist net (nigth) Nobre RA & Rosas PFC. 3♀. [CEMT]. São Miguel Arcanjo P[arque] E[stadual] Carlos Botelho 24°3′57″S 47°58′51″W 808 m 29-I-2012 Human faec. Bovy E. 1♂. [CEMT]; same data but, [24°3′47″S 47°58′45″W 823 m]. 1♀. [CEMT]. Serra do Japi 23°14′S 46°56'W Floresta 1050 m 1998 Armadilha pitfall com fezes Hernández MIM. 1♀. [CEMT].

#### Diagnosis

This species can be distinguished from the other members of the *D*. *depressicollis* species group by the following combination of characters: both sexes with pronotal process with the axes formed between their tips forming a cross (†) (Fig. 8b, e), elytra with striae distinctly impressed, shagreened, and regularly interrupted by shallow punctures (Fig. 9b), metaventrite near to the insertion of the metacoxa with denser and longer setae (Fig. 9a), metatrochanter with setae (Fig. 9a), morphology of the male genital organ as in Fig. 9c–g, and shape of endophallites (Fig. 9h-k).

#### Redescription

**Males**: Length 16.7–26.4 mm; width 8.8–15.8 mm (Fig. 8a). **Pronotum**: On the top of the pronotum in the central area has a central process as long as wide forming a cross (+) with its axes, with three pronounced points and the central region lower (Fig. 8a-–c). Surface of the pronotal disk with large and deep punctures presenting microsculpture inside (seen at high magnification) separated less than their diameter, dense in the posterocentral region of the pronotal disk and decreasing toward the apex of the pronotal protuberances where they are separated twice their diameter. **Metaventrite**: Postero-central region of metaventrite near to insertion of metacoxa with denser and longer setae (Fig. 9a). **Elytra**: Striae bicarinate, with striae distinctly impressed, shagreened, and regularly interrupted by shallow punctures (Fig. 9b). Surface of interstriae with shallow wavy appearance in all extensions, with deep and distinct punctures, separated by two to three times their diameter and visible under low magnification (Fig. 9b). **Abdomen**: fourth and fifth ventrite without setae laterally.

#### Aedeagus

Parameres are triangular with a wide apex in lateral view (Fig. 9c). In dorsal view, parameres are symmetrical; with a widened base, decreasing in thickness toward the apical region ending in a rounded tip with short setae of equal length in all extensions (Fig. 9f). Left paramere with subgenital plate partially visible (Fig. 9d). In ventral view, parameres are broad at the basal region with an acute prolongation; the apical region is narrow with a rounded apex without setae (Fig. 9g). A subgenital plate is present, broad, protruding beyond the paramere on the right side (Fig. 9e). **Endophallites**: ME is large and subquadrangular with two well sclerotized processes on the right lateral side, the surface covered with bristles along its entire length, and on the left side, it presents a membranous process with long bristles and with the spine toward the superior right edge (Fig. 9k). A+SA complex is sclerotized in the central area surrounded by a semi-sclerotized membrane (Fig. 11i). SRP has a “C” shape (Fig. 9h). FLP has an “N” shape (Fig. 9j).

#### Variation

In males, the cephalic process varies in length from 0.3 to 1.1 mm. **Females**: the length varies from 19.9 to 24.0 mm and the width from 11.3 to 14.1 mm; the cephalic process varies in length from 0.2 to 1.0 mm. In the pronotum, the central process is rounded at the anterior margin (Fig. 9d–f). Sixth abdominal ventrite is not compressed toward the middle region.

#### Distribution and Ecology

Argentina in the Misiones province and Brazil in the states of Espírito Santo, Mato Grosso do Sul, Minas Gerais, Paraná, Rio de Janeiro, Rio Grande do Sul, Santa Catarina and São Paulo (Fig. 3). It was collected in the Brazilian Cerrado, riparian forest and Atlantic Forest. From September to June, between 100 and 1334 m.a.s.l., using pitfall traps baited with human, cow, pig or carcasses; mist net and black light trap.

5. ***Dichotomius (Dichotomius) inca*** **Arias-Buriticá & Vaz-de-Mello sp. nov**

http://zoobank.org/urn:lsid:zoobank.org:act:0486A6C5-202B-4A62-8FBE-10C2728656D1

(Figs. 10a–f, 11a–k, 3 hexagons)

#### Type Material

**Holotype: **1♂. Labels: 1: {handwritten text on white label} **PERU**: **Cusco**: Machu Picchu I-2008 Condé P./2: {printed and handwritten text on red label} **HOLOTYPE** *Dichotomius inca* sp. nov. Arias-Buriticá & Vaz-de-Mello, 2026 [ex CEMT to be deposited in MUSM]./3: {printed text on white label with black margins} QR code CEMT CUIABÁ 00114578. **Paratypes**: **PERU**: Transecto Altitudinal Cusco P[uer]to Maldonado **Cuzco**: Wina Waina 15-I-1999 Etonti. 1♂. [CEMT]. **Junín**: Sanibeni 10-X-1935. 1♀. [CEMT]. Puerto Ocopa 11°10′12″S 74°18′29″W 692 m 30.iv-03.v.2012 I. Galindo & C. Espinoza. 1♂ 1♀. [MUSM]. All Paratypes have a second label: 2: {printed text on yellow label with black margins} *Dichotomius inca* sp. nov. Arias-Buriticá & Vaz-de-Mello, 2026 [♂ or ♀] **PARATYPE**.

#### Diagnosis

This species can be distinguished from the other members of the *D*. *depressicollis* species group by the following combination of characters: males with pronotal process with the axes formed between their tips forming a cross (†) and females forming a plus sign (+) (Fig. 10), elytra with striae distinctly impressed but shallow, shagreened, and regularly interrupted by shallow punctures (Fig. 11b), surface of interstriae with smooth appearance principally in the apical region; with small punctures (Fig. 11b), metaventrite near to the insertion of metacoxa without setae (Fig. 11a), metatrocanther without setae (Fig. 11a), morphology of the male genital organ as in Fig. 11c-g, and shape of endophallites (Fig. 11h-k)

#### Etymology

The specific epithet refers to the name of the Inca civilization, where the holotype was collected in one of its most famous settlements, Machu Picchu.

#### Description

**Male**: Length 26.3 mm; width 16.4 mm (Fig. 10a). On the top of the pronotum in the central area, which reaches to the medial area, it has a central process forming a cross (†) with its axes, with three pronounced points and the central one with one lobe with triangular appearance (Fig. 10a–c). Surface of the pronotal disk with large and deep punctures presenting microsculpture inside (seen at high magnification) separated less than their diameter, dense in the posterocentral region of the pronotal disk and decreasing toward the apex of the pronotal protuberances where they are separated twice their diameter. **Metaventrite**: Postero-central region of metaventrite near to insertion of metacoxa without setae (Fig. 11a). **Elytra**: Striae bicarinate, with striae distinctly impressed but shallow, shagreened, and regularly interrupted by shallow punctures (Fig. 11b). Surface of interstriae with smooth appearance principally in the apical region; with small punctures spaced twice their diameter, decreasing in depth towards the apical region (Fig. 11b). **Abdomen**: third to fifth ventrites without lateral setae.

#### Aedeagus

Parameres triangular in lateral view (Fig. 11c). In dorsal view, parameres symmetrical; with a widened base, decreasing in thickness towards apical region ending in a rounded tip with short setae of equal length in all extensions (Fig. 11f). Left paramere with subgenital plate partially visible (Fig. 11d). In ventral view, parameres broad at basal region with an acute prolongation; the apical region is narrow with rounded apex without setae (Fig. 11g). A subgenital plate is present, broad, protruding beyond the paramere on the right side (Fig. 11e). **Endophallites**: ME large and subquadrangular with two well sclerotized processes on the right lateral side, the surface covered with bristles along its entire length, and on the left side, it presents a membranous process with long bristles and with the spine in a central position (Fig. 11k). A+SA complex is sclerotized in the central area surrounded by a semi-sclerotized membrane (Fig. 11i). SRP has a “C” shape (Fig. 11h). FLP has an “N” shape (Fig. 11j).

#### Variation

In males, length varies from 22 to 23.3 mm and the width from 114 to 4.6 mm; the cephalic process varies in length from 0.8 to 1.1 mm. **Females**: the length varies from 25 to 25.8 mm and the width from 15 to 15.3 mm; the cephalic process varies in length from 0.4 to 0.6 mm. Pronotal process reaches to the third anterior region, it has a central process forming a plus sign (+) with its axes, the anterior region and the central process are rounded at the anterior margin (Fig. 10a–c). Sixth abdominal ventrite is not compressed toward the middle region.

#### Distribution and Ecology

Peru in Cuzco and Junín departments (Fig. 3). It was collected in January, April, May, and October, between 690 and 2500 m.a.s.l. There is no information about the collection method.

6. ***Dichotomius (Dichotomius) melzeri*** **(Luederwaldt, **1922**)**

(Figs. 12a–f, 13a–k, 3 squares)

*Pinotus melzeri* Luederwaldt 1922 (original description)

#### Material Examined

**Lectotype** [♂, here designated]: Labels: 1: {handwritten text on cream label with black margins} ♂/2: {printed and handwritten text on cream label with black margins} BRAZIL Rio Madeira Manicore VI.1921 Fass 2 J.F. Zikán/3: {printed text on brown label with black margins} Typus/4: {printed text on cream label} Coleção J. F. Zikan/5: {handwritten text on cream label} Pinotus ♂ Melzeri n. sp. Luederw. det.21/6. {handwritten text on cream label}Pinotus melzeri Ldw [FIOC]. **Paralectotype** [♀]: Labels: 1: {printed and handwritten text on white label with black margins} São Paulo Avanhadava 18.569/2: {handwritten text by Luederwaldt on white label} *Pinotus melzeri* ♀ Lüd. Lüd. det. 22./3: {printed text on red label with black margins} COTIPO/4: {printed text on white label} 17,157/5: {rounded and blue label} [MZSP].

Luederwaldt (1922) mentions two specimens in the original description of *Pinotus melzeri*. One specimen is a large male, from Manicoré, Rio Madeira (Amazonas, Brazil) deposited in the Zikán collection. According to Horn et al. (1990), Zikan’s collection was deposited at the Instituto Oswaldo Cruz (FIOC, Rio de Janeiro, Brazil) in 1952. FZVM found this specimen, and we here designate it as the lectotype. The paralectotype is a female specimen from Avanhandava, São Paulo, Brazil, deposited in the Museu Paulista (now the MZSP), that belongs to *D*. *barberoi* **sp. nov.** which is described in this paper.

#### Non Type Specimens

**BOLIVIA**: **La Paz**: Provincia Iturralde Rio Nuanu 12°33′95.3″S; 67°42′32.9″W 22–24-IX-2002 W. Aliaga. 1♀. [MHNNKM]. Provincia Iturralde Camp[amento] Palma Roca 23/10/1998 C. Jordan. 1♀. [MHNNKM]. **Pando**: Provincia Manuripi Florida 12°18′S; 68°40′W 187 m 03-XI-2004 C. Hamel & D. Aguirre. 1♂ 1♀. [MHNNKM]; same data but, 05-XI-2004. 2♂ 1♀. [MHNNKM]. Provincia Manuripi Santa Rosa 12°00′S; 68°52′W 182 m 23-X-2004 C. Hamel & D. Aguirre. 1♀. [MHNNKM]; same data but, 26-X-2004. 1♂. [MHNNKM]; same data but, 27-X-2004. 1♂ 1♀. [MHNNKM]. Provincia Nicolas Suárez Rutina 25-V-1999 O. Teran & D. Mamani. 1♂. [MHNNKM]. **Santa Cruz**: Provincia Angel Sandoval Murcielagos 07-V-1997 I. García. 1♂ 4♀. [MHNNKM]; same data but, 29-IV-1997. 1♀. [MHNNKM]. Provincia Urubicha 27-IV-2002 P. Coro & N. Yubanore. 1♀. [MHNNKM]. Provincia GuarayosPerseverancia 13-XI-1990 P. Bettella. 1♂. [MHNNKM]. Provincia Ñuflo de Chávez 14°44′S; 62°47′W 17-III-1990 P. Bettella. 1♀. [MHNNKM]; same data but, 18-III-1990. 1♀. [MHNNKM]; same data but, 19-III-1990. 1♂ 1♀. [MHNNKM]. Provincia Velasco Caparú 14-XI-2007 J. Ledezma et al. 7♂ 7♀. [MHNNKM]. Provincia Velasco Caparú 22-II-2007 A. Alcoba. 1♀. [MHNNKM]. Provincia Velasco Caparú 29-I-2006 A. Alcoba & M. Saldias. 3♂ 1♀. [MHNNKM]; same data but, 23-II-2006. 1♂ 1♀. [MHNNKM]; same data but, 18-VIII-2006. 1♀. [MHNNKM]; same data but, 27-XII-2006. 3♂ 5♀. [MHNNKM]; same data but, 20-II-2007. 1♂. [MHNNKM]; same data but, 22-II-2007. 4♂ 3♀. [MHNNKM]; same data but, 23-II-2007. 1♂ 4♀. [MHNNKM]; same data but, 20-III-2007. 2♂ 2♀. [MHNNKM]; same data but, 21-III-2007. 1♂ 3♀. [MHNNKM]; same data but, 22-III-2007. 3♂ 1♀. [MHNNKM]; same data but, 23-III-2007. 1♀. [MHNNKM]; same data but, 24-III-2007. 3♂ 2♀. [MHNNKM]; same data but, 20-IV-2007. 1♂ 4♀. [MHNNKM]; same data but, 21-IV-2007. 1♂. [MHNNKM]. Provincia Velasco Caparú-Lagunitas 18-VIII-2007 R. Palomino et al. 1♀. [MHNNKM]; same data but, 16-III-2008. 1♀. [MHNNKM]; same data but 04–12-VI-2007. 1♀. [MHNNKM]; same data but, 11–12-VI-2007. 6♂ 6♀. [MHNNKM]. Provincia Velasco Huanchaca 13-XI-2001 V. Chávez. 1♀. [MHNNKM]. Provincia Velasco Huanchaca 22-V-1994 S. Aysama. 1♀. [MHNNKM]. Provincia Velasco Lagunitas 18-IV-2007 A. Alcoba. 3♂ 2♀. [MHNNKM]; same data but, 1♂ 1♀. [MHNNKM]; same data but, 23–27-XII-2007. 1♀. [MHNNKM]. Provincia Velasco Lagunitas 25-I-2006 A. Alcoba & M. Saldias. 1♂. [MHNNKM]; same data but, 23-II-2006. 2♂ 1♀. [MHNNKM]; same data but, 12-IV-2006. 2♂ 1♀. [MHNNKM]; same data but, 30-XII-2006. 1♀. [MHNNKM]; same data but, 19-III-2007. 1♂ 1♀. [MHNNKM]; same data but, 22-III-2007. 1♂ 1♀. [MHNNKM]; same data but, 23-III-2007. 9♂ 9♀. [MHNNKM]; same data but, 17-IV-2007. 2♂. [MHNNKM]; same data but, 19-IV-2007. 2♂ 5♀. [MHNNKM]; same data but, 24-II-2007. 1♀. [MHNNKM]; same data but, 23-XII-2007. 1♀. [MHNNKM]. Provincia Velasco Los Fierros I-1997 B. Gill et al. 8♂ 10♀. [MHNNKM]. Provincia Velasco P[arque] N[acional] Noel Kempff Mercado 01-XI-1993 P. Bettella. 1♂. [MHNNKM]. Provincia Velasco P[arque] N[acional] Noel Kempff Mercado 06-IV-1992 J. Justiniano. 1♀. [MHNNKM]; same data but, 09-IV-1992. Provincia Velasco R[eserva] P[rivada del] P[atrimonio] N[atural] Caparú 14°48'S; 61°10'W 180 m 11-XII-2005 C. Hamel. 6♂ 9♀. [MHNNKM]; same data but, 13-XII-2005. 8♂ 10♀. [MHNNKM]; same data but, 19-XII-2005. 1♀. [MHNNKM]. **BRAZIL**: **Acre**: sec. For Rio Branco PZ UFAC II-1997 Vaz-de-Mello. 2♂. [CEMT]. Rio Branco Fazenda Catuaba II-1997 Vaz-de-Mello FZ. 2♀. [CEMT]. Senador Guiomard 10°4′S 67°37′W 14-IV-2017 fezes humanas Bitencourt B. 1♂ 1♀. [CEMT]. Xapuri Reserva Chico Mendes 10°28′25″S 68°41′60″W Floresta Amazônica 25-X-2008 pitfall com fezes humanas Silveira J. 1♀. [CEMT]. **Amazonas**: BR 319 km 350 5°12′56.4″S 61°50′22.6″W 30-VII-2-VIII-2007 Gasca H. 2♂ 1♀. [CEMT]. **Maranhão**: Centro Novo do Maranhão ReBio Gurupi 3°15′0.4″S 46°40′43″W 02–04.ix.2022 hum. Feces E. Lucena & E. Carvalho. 2♀. [CEMT]; same data but, [3°15′0.7″S 46°45′25″W 01–03.ix.2022]. 1♂ 1♀. [CEMT]; same data but, [3°20′22″S 46°45′19″W 04–06.ix.2022]. 1♂ 1♀. [CEMT]. **Mato Grosso**: Alta Floresta Ceplac 06-II-2009 FIT Gonçalves V. 1♀. [CEMT]; same data but, [5-III-2010]. 1♂. [CEMT]. Alta Floresta fragmento 16 9°41′24″S 55°56′28″W VI-2008 pitfall c/hum faec Berenguer E. 2♂ 3♀. [CEMT]; same data but, [Fragmento 26 9°53′41″S 56°16′35″W]. 8♂ 10♀. [CEMT]; same data but, [Fragmento 27 9°49′44″S 56°19′51″W]. 13♂ 6♀. [CEMT]; same data but, [Fragmento 29 9°56′52″S 56°3′2″W]. 1♀. [CEMT]; same data but, [Fragmento 48 9°58′46″S 56°5′31″W]. 4♂ 5♀. [CEMT]; same data but, [fragmento 67 9°46′22″S 55°11′48″W]. 2♀. [CEMT]; same data but, [Fragmento 94 9°51′59″S 55°54′2″W]. 1♂ 1♀. [CEMT]; same data but, [Fragmento 114 9°45′19″S 55°58′19″W]. 3♂ 6♀. [CEMT]; same data but, [Fragmento 145 9°51′6″S 55°59′4″W]. 2♂. [CEMT]; same data but, [Fragmento 150 9°35′54″S 55°56′9″W]. 2♂ 1♀. [CEMT]; same data but, [Fragmento 151 9°44′55″S 56°1′41″W]. 2♂. [CEMT]; same data but, [Fragmento 153 9°50′22″S 56°0′21″W]. 1♂ 1♀. [CEMT]; same data but, [Fragmento 155 10°1′13″S 56°22′11″W]. 2♂ 1♀. [CEMT]; same data but, [Fragmento FAH 9°52′53″S 56°6′13″W]. 1♂. [CEMT]. Alta Floresta P[arque] E[stadual] Cristalino 9°32′45″S 55°54′56″W 248 m 15–17-XI-2019 hum dung Azevedo RA Plot A P06 (1100). 2♀. [CEMT]; same data but, [Plot A. P07 (1200)]. 1♀. [CEMT]; same data but, [Plot A. FIT-1 (7500)]. 1♂. [CEMT]; same data but, [Plot A. P10 (1500)]. 1♀. [CEMT]; same data but, [Plot B. P01 (600)]. 1♂. [CEMT]. Aripuanã 10°3′10''S 59°29′42″W 320 m 26-I-2012 Faria HAB. 1♂ 1♀. [CEMT]. Carlinda 14-V-2008 Ortíz ME. 1♂. [CEMT]; same data but, [15-V-2008 Francisco ME]. 1♀. [CEMT]. Cotriguaçu Fazenda São Nicolau 15-XII-2007 Peres-Filho O. 1♂. [CEMT]. Cotriguaçu V-2011 Vicente RE. 1♀. [CEMT]. Cotriguaçu Fazenda São Nicolau V-2008 luz Peres-Filho O. 1♂ 1♀. [CEMT]. Cotriguaçu Fazenda São Nicolau Castanheira 9°49′18″S 58°17′18″W 2009 Pitfall fezes 48 h Vaz-de-Mello FZ. 1♀. [CEMT]; same data but, [prainha 9°51′36″S 58°12′53″W 11–13-XII-2009]. 2♂. [CEMT]; same data but, [matinha-borda da mata 9°50′19″S 58°15′3″W 13-XII-2009]. 1♂. [CEMT]; same data but, [mata norte 9°49′9″S 58°15′47″W 08–10-XII-2009]. 1♀. [CEMT]; same data but, [9°50′53″S 58°14′36″W 11-XII-2009]. 1♀. [CEMT]. Cotriguaçu Fazenda São Nicolau Prainha 9°51′36″S 58°12′53″W 11-XII-2009 rede de neblina Oliveira DM. 1♀. [CEMT]. Cotriguaçu Fazenda São Nicolau Talhão 49a[nos] 9°51′16.72″S 58°14′13.75″W 1-XI-2010 pitfall fezes hum. Gigliotti MS. 1♂ 1♀. [CEMT]. Cotriguaçu Fazenda São Nicolau 9°49′17″S 58°15′32″W 01-XI-2017 FIT Vaz-de-Mello FZ. et al. 1♀. [CEMT]. Cotriguaçu Fazenda São Nicolau 9°51′18″S 58°16′14″W 22-VIII-2019 hum. Feces Maldaner ME & Manjate NM. 1♀. [CEMT]. Cotriguaçu Fazenda São Nicolau 9°51′18''S 58°16′19″W 04–06-XI-2017 Pitfall human feces Santos J. P. 1♀. [CEMT]; same data but, [secondary forest 9°50′40″S 58°14′54″W]. 2♀. [CEMT]; same data but, [primary forest 9°49′14″S 58°15′31″W]. 1♀. [CEMT]. Cotriguaçu Fazenda São Nicolau 09°52′10″S 58°15′40″W Pitfall fezes humanas 18–20-X-2021 Mariano et al. 1♂ 2♀. [CEMT]; same data but, [09°52′05″S 58°16′04″W]. 1♂. [CEMT]; same data but, [09°49′47″S 58°16′19″W 01–03-XI-2021]. 1♂. [CEMT]; same data but, [09°49′47″S 58°16′26″W 01–03-XI-2021]. 1♂. [CEMT]; same data but, [09°51′32″S 58°14′23″W 02–04-XI-2021]. 1♂ 1♀. [CEMT]; same data but, [09°51′26″S 58°14′24″W 02–04-XI-2021]. 1♀. [CEMT]; same data but, [09°49′43″S 58°16′30″W 03–05-XI-2021]. 1♀. [CEMT]; same data but, [09°49′49″S 58°16′08″W 04–06-XI-2021]. 2♂ 2♀. [CEMT]; same data but, [09°49′49″S 58°16′09″W 04–06-XI-2021]. 1♀. [CEMT]; same data but, [09°50′26″S 58°15′10″W 04–06-XI-2021]. 1♀. [CEMT]; same data but, [09°50′29″S 58°15′06″W 04–06-XI-2021]. 1♀. [CEMT]; same data but, [09°50′58″S 58°15′23″W 04–06-XI-2021]. 1♀. [CEMT]; same data but, [09°51′11″S 58°16′22″W 05–07-XI-2021]. 2♂ 3♀. [CEMT]; same data but, [09°51′41″S 58°15′50″W 05–07-XI-2021]. 1♂ 2♀. [CEMT]; same data but, [09°51′45″S 58°15′50″W 05–07-XI-2021]. 1♀. [CEMT]; same data but, [09°50′57″S 58°14′03″W 05–07-XI-2021]. 2♀. [CEMT]; same data but, [09°49′30″S 58°16′12″W 05–07-XI-2021]. 2♀. [CEMT]; same data but, [09°49′29″S 58°16′14″W 05–07-XI-2021]. 1♂. [CEMT]; same data but, [09°49′27″S 58°16′14″W 05–07-XI-2021]. 1♂. [CEMT]; same data but, [09°49′32″S 58°16′39″W 05–07-XI-2021]. 1♀. [CEMT]; same data but, [09°49′29″S 58°16′39″W 05–07-XI-2021]. 1♂. [CEMT]; same data but, [09°50′58″S 58°15′23″W 04–06-XII-2021]. 1♀. [CEMT]. Diamantino 16-II-2018 Nunes & Carvalho. 1♂. [CEMT]. Novo Mundo P[arque] E[stadual] Cristalino 9°28′1″S 55°47′43″W V-2013 pitfall Magalhães V. 1♀. [CEMT]; same data but, [9°27′53″S 55°49′30″W]. 1♀. [CEMT]; same data but, [9°27′60″S 55°50′2″W]. 1♀. [CEMT]; same data but, [Novo Mundo P[arque] E[stadual] Cristalino Manual Corrêa VS]. 1♂. [CEMT]. Novo Mundo P[arque] E[stadual] Cristalino 9°27′33″S 55°47′59″W 18-IV-2021 Hum[an] dung Rodrigues D. J. 2♀. [CEMT]; same data but, [9°27′55″S 55°49′34″W]. 1♂ 1♀. [CEMT]; same data but, [9°28′2″S 55°50′6″W]. 3♂. [CEMT]; same data but, [9°28′8″S 55°50′41″W]. 2♂ 1♀. [CEMT]; same data but, [9°38′32″S 55°20′36″W]. 1♂. [CEMT]; same data but, [9°37′34″S 55°20′39″W]. 1♂. [CEMT]; same data but, [9°29′43″S 55°12′52″W 19-IV-2021]. 1♀. [CEMT]; same data but, [9°30′59″S 55°12′6″W 19-IV-2021]. 1♀. [CEMT]; same data but, [9°30′9″S 55°12′38″W 19-IV-2021]. 1♂. [CEMT]; same data but, [9°31′25″S 55°11′48″W 19-IV-2021]. 1♂ 4♀. [CEMT]. Paranaita 9°15′45″S 56°49′5″W XII-2015 pitfall Bragança MAL. 1♂. [CEMT]. Paranaita Rio Teles Pires Fazenda Aragão 9°13′37″S 56°59′56″W margem esquerda 15-VI-2009 Pitfall com fezes humanas Souza MF. & Uehara-Prado M. 1♀. [CEMT]. Paranaita Modulo Paranaita 9°32′52.4″S 56°42′5.3″W 5-V-30-VI-2009 Ribeiro RK. 1♂. [CEMT]. **Pará**: Belterra 735988 9668191 260_t6 15-VII-2016 pitfall fezes hum/suinas França F. 1♀. [CEMT]; same data but, [734436 9634987 357_t3]. 1♂. [CEMT]. Novo Progresso Fazenda Florentino 7°8′57″S 55°23′41″W 229 m Floresta amazónica I-2011 pitfall-cow dung Pelissari. 1♂ 1♀. [CEMT]; same data but, [7°6′50''S 55°25′24″W 217 m]. 1♀. [CEMT]; same data but, [7°07′21″S 55°25′13″W 213 m pasto III-2011]. 1♂. [CEMT]. Pau d’Arco Fazenda Marajoara 7°50′S 50°16′W 13-X-1998 Scheffler PY. 1♂ 1♀. [CEMT]. Redenção Pinkaiti-Aik 7°44′S 52°2′W VI-1999 Scheffler PY. 1♀. [CEMT]; same data but, [7°46′S 51°58′W XI-1999]. 1♂. [CEMT]. Santarém XI-1997. 1♂. [CEMT]. Santarém Amaz[on] III-1923 boy Zikán JF. 1♀. [CEMT]. Santarém Reseva Tapajós 2°36′513″S 55°36′513 W Floresta Amazônica 350 m 7-I-2009 pitfall com fezes humanas Andrade R. 1♂ 1♀. [CEMT]; same data but, [2°36′529″S 55°36′421″W]. 1♂. [CEMT]. Santarém Usina Hidrelétrica de Curuá-Una 2°48′16″S 54°15′23″W 23-VI-2018 human dung Ganança & Vasconcelos-Neto. 2♀. [CEMT]; same data but, [2°49′15″S 54°17′46″W]. 1♂. [CEMT]; same data but, [2°47′47″S 54°17′42″W]. 2♀. [CEMT]; same data but, [2°48′41″S 54°18′3″W]. 2♀. [CEMT]; data but, [2°48′51″S 54°17′4″W]. 2♀. [CEMT]; same data but, [2°48′20″S 54°17′44″W 22-VI-2018]. 1♀. [CEMT]; same data but, [2°48′35″S 54°17′34″W 22-VI-2018]. 1♀. [CEMT]; same data but, [2°48′35″S 54°18′16″W 22-VI-2018]. 1♂ 1♀. [CEMT]; same data but, [2°48′57″S 54°16′59″W]. 1♂. [CEMT]; same data but, [2°49′15″S 54°17′39″W 22-VI-2018]. 3♀. [CEMT]; same data but, [2°48′31″S 54°18′18″W 22-VI-2018]. 1♂ 4♀. [CEMT]; same data but, [2°48′16″S 54°18′23″W 22-VI-2018]. 1♀. [CEMT]; same data but, [2°48′57″S 54°16′59″W 22-VI-2018]. 1♀. [CEMT]. São Felix do Xingu Pinkaití Reserve 7°45′S 51°57′W Forest 31-X-1998 Scheffler PY. 1♀. [CEMT]. Tailándia Empresa Agropalma 09–15-VII-2016 Silva F. 1♂. [CEMT]. **Rondônia**: 62 km S Ariquemes Fazenda Rancho Grande XI-1996 Vulinec & Mellow. 1♂ 1♀. [CEMT]. Alto Paraiso Rio Candeias IX.2003 Fam[ilia] W. Furt leg. 1♂ 2♀. [CEMT]; same data but, [X.2002]. 1♀. [CEMT]. Alto Paraiso Rio Candeias 13–23-IV-2013 Furtado E. 1♂ 1♀. [CEMT]. Cacoal Fazenda agroecológica 11°31′44″S 61°34′15″W floresta 19-III-2017 pitfall hum dung Silva R. 3♂. [CEMT]. Chupinguaia 12°13′30″S 60°43′22″W 2016 Semedo T. & Ribeiro. 1♀. [CEMT]. Guajará-Mirim Fazenda Estrela de Davi 10°44′35.17″S 65°17′58.57″W 165 m floresta Amazônica 18-II-2010 isca:fez hum Coletti F. 1♂ 1♀. [CEMT]. Itapuã do Oeste Flo[resta] Na[cional] Jamari 9°5′19″S 63°9′38″W 20-II-2013 *Mazama gouazoubira* feces Cerveira JF. 1♂. [CEMT]. Porto Velho ES[tação] EC[ologica] Cuniã 8°4′11.82″S 63°28′34.64″W 83 m 10–12-XI-2013 Pitfall human dung Silveira MAPA. 9♂ 6♀. [CEMT]. Porto Velho Abunã 9°35′3″S 65°22′36″W 30-VI-2010 pitfall human feces Falcão JCF. & Silva RLR. 1♀. [CEMT]. Porto Velho Nova Mutum Parana T3P2A3 9°26′52″S 61°9′56″W 01–03-III-2010 fezes hum. Silva LR. & Silva RLR. 1♂ 1♀. [CEMT]. Rolim de Moura 11°44′4.93″S 61°55′9.19''W 215 m-SAF 08–10-XII-2015 Pitfall human dung Castro D.C. leg. 2♂ 5♀. [CEMT]; same data but, [11°36′4.41″S 61°52′9.15″W]. 1♂. [CEMT]; same data but, [11°44′3.33″S 61°55′11.53″W 223 m]. 1♂ 1♀. [CEMT]; same data but, [11°44′3.83″S 61°55′10.97″W 220 m]. 1♂. [CEMT]; same data but, [11°44′4.42″S 61°55′11.59″W 220 m]. 4♂ 2♀. [CEMT]; same data but, [11°44′4.48″S 61°55′12.75″W 222 m]. 1♂. [CEMT]; same data but, [11°44′4.48″S 61°55′12.14″W 221 m]. 1♀. [CEMT]; same data but, [11°44′4.87″S 61°55′8.64″W 214 m]. 1♀. [CEMT]; same data but, [11°44′4.94″S 61°55′9.80″W 216 m]. 2♂. [CEMT]; same data but, [11°44′5.00″S 61°55′11,62″W 218 m]. 1♀. [CEMT]; same data but, [11°44′5.02″S 61°55′12.19″W 220 m]. 1♂ 2♀. [CEMT]; same data but, [11°44′24.03″S 61°55′26,26″W 275 m 07–09-XII-2015]. 1♂. [CEMT]; same data but, [11°44′24.07″S 61°55′26,79''W 275 m 07–09-XII-2015]. 1♀. [CEMT]; same data but, [11°44′25.07″S 61°55′27,36″W 275 m 07–09-XII-2015]. 1♂. [CEMT]. **PERU: Madre de Dios**: Puerto Maldonado Sudadero 12°21′19’’S 69°1′48’’W 21–22.vii.2009 221 m M. Alvarado. 2♀. [MUSM]; same data but, 12°21′57″S 69°4′15″W 18–19.vii.2009. 1♀. [MUSM]; same data but, [12°21′57″S 69°4′18″W 18–19.vii.2009]. 1♀. [MUSM]. Puerto Maldonado Sudadero 12°21′19’’S 69°1′48″W 27.iii.2009 221 m L. Figueroa. 2♂. [MUSM]; same data but, [12°21′57″S 69°4′18″W 28.iii.2009]. 1♀. [MUSM]. Fundo Vivero El Bosque 233 m 12°27′49.27″S 69°7′30.69″W 17–19.iv.2011 O. Huaches. 2♂ 2♀. [MUSM]. Río Madre de Dios Río Los Amigos base camp 1° terra firme 290 m pitfall human dung 12°34′10″S 70°06′1.4″W iv.2000 T. Larsen. 1♂ 1♀. [MUSM]. Patuyacu Oculto camp 12°39′00″S 68°55′33″W Pitfall human dung 400 m II-8–1999 T. Larsen. 1♂. [MUSM]. Tambopata R[eserva] N[atural] Tambopata Refugio Amazonas Lodge 12°52′55.2″S 69°25′7.9″W 23–25.v.2015 J. Peralta & P. Sánchez. 1♂. [MUSM]. Río Los Amigos (Cicra) 280 m 12°34’S 70°05′W 10–14.vii-2010 C. Chaboo. 1♀. [MUSM]. Manu Los Amigos 12.5691667S 70.10011°W 300 m 24.iv-3.v.2017 K. Siu Ting. 1♂. [MUSM]. Tambopata Albergue Posada Amazonas 12°48′21″S 69°17′23″W 151 m 10–18.ii.2012 J. Peralta & E Rázuri. 1♀. [MUSM]. Río Tambopata Res[erva]. 30Air km. SW Pto Maldonado 290 m 11–15.XI.1979 JB Heppner subtropical moist forest. 1♀. [MUSM]. Tambopata Las Piedras Comunidad Alegrías 12°0′26.13″S 69°11′4.81″W 227 m 6–11.iii.2022 A. Ayala & L. Inga. 1♂ 1♀. [MUSM]. Tambopata 12°42′39.51S 69°13′47.63″W 219 m 16.vi.2019 C. Torres. 1♂ 1♀. [MUSM]. **Puno**: Sandia San Pedro de Putina Punco P[arque] N[acional] Bahuaja Sonene 13°23′31.1″S 69°28′54.4″W 325 m 11–24.ix.2011 E. Guillermo & E. Rázuri. 1♀. [MUSM]; same data but, [13°23′24″S 69°29′2.9’’W 328 m]. 1♂ 1♀. [MUSM].

#### Diagnosis

This species can be distinguished from the other members of the *D*. *depressicollis* species group by the following combination of characters: both sexes with pronotal process with the axes formed between their tips forming a cross (†), rounded and wide in central region, surface of pronotal process with large circular to elongate punctures spaced less than one times their size and joined in some places, giving the appearance of a wavy surface (Fig. 12), elytra with striae distinctly impressed, shagreened, and regularly interrupted by shallow punctures (Fig. 13b), metaventrite with setae on the anterior surface reaching to the central region of the mesocoxae (Fig. 13a), metatrocanther without setae (Fig. 13a), morphology of the male genital organ as in Fig. 13c-g, and shape of endophallites (Fig. 13h-k).

#### Redescription

**Male**: Length 18.2–28.3 mm; width 11.1–16.9 mm (Fig. 12a). On the top of the pronotum in the central area, which reaches to the anterior third area, it has a central process forming a cross (†) with its axes, with three pronounced points and the central one with one lobe with a wide triangular appearance (Fig. 12a–c). Surface of the pronotal disk with large and deep punctures presenting microsculpture inside (seen at high magnification) separated less than their diameter, dense in the posterocentral region of the pronotal disk and forming wrinkles toward the apex of the pronotal protuberances. **Metaventrite**: Anterior surface with dense setae on the reaching to the central region of the mesocoxae; some individuals may have a few setae or a line of setae, but never abundant (Fig. 13a). **Elytra**: Striae bicarinate, with striae distinctly impressed, deep, shagreened, and regularly interrupted by shallow punctures (Fig. 13b). Surface of interstriae with a smooth appearance principally in the apical region; with small punctures spaced twice their diameter, decreasing in depth towards the apical region (Fig. 13b). **Abdomen**: third to fifth ventrites without lateral setae.

#### Aedeagus

Parameres triangular with acute apex in lateral view (Fig. 13c). In dorsal view, parameres asymmetrical, the right one being slightly shorter; with a widened base, decreasing in thickness towards the apical region ending in a rounded tip with short setae of equal length in all extensions (Fig. 13f). Left paramere with subgenital plate partially visible (Fig. 13d). In ventral view, parameres broad in the basal region with an acute prolongation; the apical region is narrow with rounded apex with short setae (Fig. 13g). A subgenital plate is present, broad, protruding beyond the paramere on the right side (Fig. 13e). **Endophallites**: ME large and subquadrangular with two well sclerotized processes on the right lateral side, the surface covered with bristles along its entire length, and on the left side it presents a membranous process with long bristles and with the spine toward the right edge (Fig. 13k). A+SA complex is sclerotized in the central area surrounded by a semi-sclerotized membrane (Fig. 13i). SRP has a “C” shape, wide, with a wide sclerotized base (Fig. 13h). FLP has an “N” shape well sclerotized (Fig. 13j).

#### Variation

In males, the cephalic process varies in length from 0.4 to 1.0 mm. **Females**: the length varies from 17.3 to 27.1 mm and the width from 10.9 to 17.4 mm; the cephalic process varies in length from 0.2 to 0.8 mm. Pronotal process reaches to the third anterior region; it has a central process forming a plus sign (+) with its axes, the anterior region and the central process are rounded at the anterior margin and with wrinkles in the surface (Fig. 12 a–c). Sixth abdominal ventrite is not compressed toward the middle region.

#### Distribution and Ecology

Bolivia in the departments of La Paz, Pandó and Santa Cruz; Brazil in the states of Acre, Amazonas, Mato Grosso, Maranhão, Pará and Rondônia and Peru in the Madre de Dios and Puno departments (Fig. 3). It was collected throughout the year in the Amazon biome, between 100 and 400 m.a.s.l., using pitfall traps baited with human and *Mazama gouazoubira* (gray brocket deer) dung, mist net, light trap and FIT.

7. ***Dichotomius (Dichotomius) salomaoi*** **Arias-Buriticá & Vaz-de-Mello sp. nov**

(Figs. 14a–f, 15a–j, 3 pentagons)

#### Type Material

**Holotype**: 1♂. Labels: 1: {printed text on white label} **BRAZIL: Paraíba**: Santa Rita Gargáu Private Reserve 7°1'S 34°57'W rainforest 19-II-2018 human feces Salomão RP./2: {printed and handwritten text on red label} **HOLOTYPE** *Dichotomius salomaoi* sp. nov. Arias-Buriticá & Vaz-de-Mello, 2026 [CEMT]/3: {printed text on white label with black margins} QR code CEMT CUIABÁ 00115213. **Paratypes**: same data of holotype. 2♂ 7♀. [CEMT]. **BRAZIL**:** Alagoas**: Ibateguara dois braços 3-XI-2007 carcaça Filgueiras BKC. 1♀. [CEMT]. **Ceará**: Crato Trilha do Belmonte 8-VII-2007 Oliveira S. & Moura R. 4♂. [CEMT]; same data but, [9-VII-2007]. 1♂. [CEMT]. Porteiras Floresta Nacional do Araripe 22-II-2014 Bovine dung Puker A. 1♂ 1♀. [CEMT]. Ubajara P[arque] N[acional] Ubajara Trilha Samambaia 3°50′26″S 40°54′22″W 825 m 19-II-2013 Pitfall hum dung Vaz-de-Mello & Grossi. 2♂ 6♀. [CEMT]. **Maranhão**: Lajeado Novo 6°9′28″S 47°2′45″W carcass Da Silva V.R. 09-IV-2022. 1♂. [CEMT]. São João do Paraíso 6°32′3″S 46°54′45″W 12-I-2021 human feces Batista M.C. 7♂ 3♀. [CEMT]; same data but, [6°30′45″S 46°56′28″W]. 1♀. [CEMT]. **Pernambuco**: Paudalho Aldeia mata atlântica 28-II-2005 pitfall com fezes humanas Silva F. et al. 5♂ 3♀. [CEMT]. **Sergipe**: Areia Branca Parque Nacional Serra de Itabaiana 10°45′48″S 37°20′22″W 25-VII-2022 Human dung Uehara-Prado M. et al. 1♀. [CEMT]. All Paratypes have a second label: 2: {printed text on yellow label with black margins} *Dichotomius salomaoi* sp. nov. Arias-Buriticá & Vaz-de-Mello, 2026 [♂ or ♀] **PARATYPE**.

#### Diagnosis

This species can be distinguished from the other members of the *D*. *depressicollis* species group by the following combination of characters: males with pronotal process with the axes formed between their tips forming a cross (†) and females forming a plus sign (+) (Fig. 14b, e), elytra with striae distinctly impressed but shallow, shagreened, and regularly interrupted by shallow punctures (Fig. 15b), surface of interstriae with shiny surface, with distinct wrinkles in all extensions, with small and deep punctures (Fig. 15b), metaventrite near to the insertion of metacoxa without setae (Fig. 15a), metatrocanther without setae (Fig. 15a), morphology of the male genital organ as in Fig. 15c-g, and shape of endophallites (Fig. 15h-k)

#### Etymology

This species is named in honor of Dr. Renato Portela Salomão in recognition of his contributions to ecology and conservation of Caatinga dung beetles, and he collected part of the type series of this species.

#### Description

**Male**: Length 25.5 mm; width 15.7 mm (Fig. 14a). On the top of the pronotum in the central area, which reaches to the medial area, it has a central process forming a cross (†) with its axes, with three pronounced points and the central one with one lobe with a triangular appearance (Fig. 14a–c). Surface of the pronotal disk with large and deep punctures presenting microsculpture inside (seen at high magnification) separated less than their diameter, dense in the posterocentral region of the pronotal disk and forming short wrinkles towards the apex of the pronotal protuberances. **Metaventrite**: Postero-central region of metaventrite near to the insertion of the metacoxa without setae (Fig. 15a). **Elytra**: Striae bicarinate, with striae distinctly impressed, deep, shagreened, and regularly interrupted by shallow punctures (Fig. 15b). Interstriae with shiny surface, with distinct wrinkles in all extensions, with small and deep punctures, separated by twice their diameter and visible under low magnification (Fig. 15b). **Abdomen**: third to fifth ventrites without lateral setae.

#### Aedeagus

Parameres triangular in lateral view (Fig. 15c). In dorsal view, parameres asymmetrical, the right one being slightly shorter; with a widened base, decreasing in thickness towards apical region ending in a rounded tip with short setae of equal length in all extensions (Fig. 15f). Left paramere with subgenital plate partially visible (Fig. 15d). In ventral view, parameres broad at basal region with an acute prolongation; the apical region is narrow with rounded apex with short setae (Fig. 15g). A subgenital plate is present, broad, protruding beyond the paramere on the right side (Fig. 15e). **Endophallites**: ME large and subquadrangular with two well sclerotized processes on the right lateral side, the surface covered with bristles along its entire length, and on the left side it presents a membranous process with long bristles and with the spine toward the right edge (Fig. 15k). A+SA complex is sclerotized in the central area surrounded by a semi-sclerotized membrane (Fig. 15i). SRP has a “C” shape, wide, with a thin line sclerotized (Fig. 15h). FLP has an “N” shape (Fig. 15j).

#### Variation

In males, length varies from 15.61 to 25.6 mm and the width from 9.5 to 16 mm; the cephalic process varies in length from 0.2 to 1.1 mm. Some minor specimens with pronotal process rounded anteriorly. **Females**: the length varies from 17 to 25.5 mm and the width from 10.7 to 17.1 mm; the cephalic process varies in length from 0.2 to 0.96 mm. Pronotal process reaches to the third anterior region, it has a central process forming a plus sign (+) with its axes, the anterior region and the central process is rounded at the anterior margin and with short wrinkles in the surface (Fig. 14d-–f). Sixth abdominal ventrite is not compressed toward middle region.

#### Distribution and Ecology

Northeastern of Brazil in the states of Alagoas, Ceará, Maranhão, Paraíba, Pernambuco, and Sergipe (Fig. 3). It was collected in January, February, April, July, and November, between 30 and 825 m.a.s.l., using pitfall traps baited with human, cow, and carcass.

8. ***Dichotomius (Dichotomius) zikani*** **(Luederwaldt, **1922**)**

(Figs. 16a–f, 17a–k, 3 asterisk)

*Pinotus zikani* Luederwaldt 1922: 4 (original description)

#### Material Examined

**Lectotype** [♂, here designated]: Labels: 1: {printed and handwritten text on white label} Mato Grosso I.99./2: {handwritten text by Luederwaldt on white label} *Pinotus zikani* ♂ Lüd. Lüd. det. 22./3: {printed on white label} 17172/4: {white label with one triangular point} [sexual organ without parameres] [MZSP]. **Paralectotype** [**♂**]: Labels: 1: {printed and handwritten text on white label} XI. Virginia S. Minas Ger. Faz. Campos 1500 m. 1920 J.Zikán/2: {handwritten text} Pinotus zikani Ldw./3: {handwritten text by Luederwaldt on white old label}*Pinotus ♂ zikani,* n. sp. i. det. Luederw. det. 21./4: {printed text on white label} Colecao J F. Zikán/5: {handwritten text on small white label}**♂/**6: {printed text on orange label with black margins} Typus. [FIOC].

In the original description of *Pinotus zikani*, Luederwaldt (1922) mentions two male specimens. The first from Mato Grosso (Brazil), deposited in the Museo Paulista [now the MZSP], is here designated as the Lectotype. We found a specimen deposited in the Zikán collection [now FIOC], that is a big male of *D*. *depressicollis*, labeled as “type” of *P*. *zikani*. Although the label says Virginia and not Passa Quatro, Luederwaldt’s handwriting is typical enough for us to consider it as a paralectotype and the wrong locality as lapsus.

#### Non Type Specimens

**BOLIVIA: Santa Cruz**: Velasco Prov[incia] Huanchaca II P[arque] N[atural] Noel Kempf Mercado 14°31′40.4″S 60°44′15″W 26–31-X-1999 780 m S. Spector. Cerrado South Transect. 1♀. [NMNH]. **BRAZIL**: **Rondônia**: Vilhena X-1966 Alvarenga M. 1♀. [CEMT]. **Mato Grosso**: Fazenda São Tiago 12°35′S 56°20′W X-[19]81. 1♂. [CEMT].

#### Diagnosis

This species can be distinguished from the other members of the *D*. *depressicollis* species group by the following combination of characters: both sexes with pronotal process with the axes formed between their tips forming a cross (†) and central process bilobate (Fig. 16), elytra with striae distinctly impressed but shallow, shagreened, and regularly interrupted by shallow punctures (Fig. 17b) and surface with blue spots (Fig. 17b), metaventrite near to the insertion of the metacoxa without setae (Fig. 17a), metatrochanter with setose punctures with long setae (Fig. 17a), morphology of the male genital organ as in Fig. 17c-g, and shape of endophallites (Fig. 17h-k).

#### Redescription

Length 23.3; width 14.5 (Fig. 16a). **Pronotum**: On the top of the pronotum in the central area, that reaches the anterior third, has a central process as long as wide that forms a plus sign (+) with its axes, with three pronounced points and the central one with two defined lobes (Fig. 16a-–c). Surface of the pronotal disk with large and deep punctures presenting microsculpture inside (seen at high magnification) separated less than their diameter, dense in the central and posterocentral region of the pronotal disk and decreasing towards the apex of the pronotal protuberances. **Metaventrite**: Postero-central region of metaventrite near to insertion of metacoxa without setae (Fig. 17a). **Elytra**: Surface with blue spots. Striae bicarinate, with striae distinctly impressed but shallow, shagreened, and regularly interrupted by shallow punctures (Fig. 17b). Surface of interstriae with smooth appearance; with small and shallow punctures and separated by three times their diameter and visible under low magnification (Fig. 17b). **Abdomen**: third to fifth ventrites without lateral setae.

#### Aedeagus

Parameres triangular in lateral view (Fig. 17c). In dorsal view, parameres symmetrical with a widened base, decreasing in thickness towards the apical region where they terminate in a truncate tip with short setae, longer on the sides (Fig. 17d, f). Left paramere with subgenital plate not visible (Fig. 17d). In ventral view, parameres broad at the basal region with an acute prolongation; the apical region is narrow with a truncate apex and without setae (Fig. 17g). Subgenital plate present, but allowing the middle region of the parameres to be seen (Fig. 17e). **Endophallites**: ME large and subquadrangular with two well sclerotized processes on the right lateral side, the surface covered with bristles along its entire length, and on the left side it presents a membranous process with long bristles and with the spine in a central position (Fig. 17k). A+SA complex is sclerotized in the central area surrounded by a semi-sclerotized membrane (Fig. 17i). SRP has a “C” shape, wide (Fig. 17h). FLP has an “N” shape (Fig. 17j).

#### Variation

Cephalic process of male with 1.3 mm. **Females**: the length varies from 24.6 to 28.9 mm and the width from 15.8 to 18 mm; the cephalic process varies in length from 1.1 to 1.3 mm. In the pronotum, the central process is similar to males (Fig. 16d-f).

#### Distribution and Ecology

Bolivia in the Santa Cruz department and Brazil in the states of Rondônia and Mato Grosso (Fig. 3). It is a rare species in entomological collections and with very few captures in field collections. It was collected in January and October, up to 780 m.a.s.l. There is no information about the collection method.

## Discussion

The taxonomic revision of the species group *D*. *depressicollis *sensu Arias-Buriticá and Vaz-de-Mello (2025) is presented here. Initially, the group contained three species and was finally expanded to eight species, of which five species are new and described in this revision. The species of this group little vary in their external morphology; however, we have managed to differentiate them and to establish characteristics that allow us to separate them, mainly from the morphology of the thorax, striae and interestriae, setae position on the metaventrite, and the male genitalia.

Among the characters that help in this correct delimitation of the species, the morphology of the pronotal process, which presents two morphologies that we have called here cross-shaped and plus-shaped, helps us to partially separate the species of the group. Then, the structure of the striae and the punctuation and microsculpture of the interstriae help to delimit the species. In addition, the morphology of the distribution of the setae in the metaventrite is considered as a relevant character to separate the species, mainly *D*. *depressicollis*, which presents complete setae in the metaventrite and allows us to easily separate it from the other species (Fig. 9a).

Regarding the morphology of the male genitalia, seven of the eight species have a subtriangular pattern of parameres with rounded apices in lateral view, with one species, *D*. *zikani*, which has a subtriangular shape in the parameres but obtuse apices, being the most different species (Fig. 17d-–g). However, the lateral shape of the parameres varies considerably in the other species, which helps to correctly delimit and separate them, as well as the asymmetry in dorsal view in three of the species of the group. The medial endophallite of this group presents a sclerotized area in the central part forming a tooth, which is an almost exclusive feature of this group (Fig. 2j, 5j, 7j, 8j, 11j, 13j, 15j, 17j), present only in only one other species of the genus within the *Dichotomius bitiensis* species group, but retained in this group because of the external morphology in which the structure of the conical cephalic process and the absence of the two sinuous subparallel carinae in the medial–lateral fovea of the pronotum differ from that of the *D*. *depressicollis* group.

The group is exclusive from South America, with three species distributed in the Dry Diagonal (*D*. *barberoi* **sp. nov.**, *D*. *davidedmondsi* **sp. nov.** and *D*. *salomaoi* **sp. nov.**), two species in the Atlantic Forest (*D*. *audinoae* **sp. nov.** and *D*. *depressicollis,* the last with some registers in Cerrado), one species in the Amazon (*D*. *melzeri*), one species in the Amazon-Cerrado transition (*D*. *zikani*) and one species in the Andes of Peru (*D*. *inca* **sp. nov.**). The species are relatively common, but with the exception of *D*. *inca* **sp. nov.** and *D*. *zikani*, are considered rare species with a few specimens in entomological collections.

For the position of the cephalic process in males and females and the pronotal morphology, the species of the *D*. *depressicollis* species group are close to the *Dichotomius carolinus* species group, but easily distinguishable by the processes that are bicuspid in the *D*. *depressicollis* species group and more conical in the *D*. *carolinus* species group; in addition to the widening of the striae that the *carolinus* group presents. For male genital morphology (shape and presence of apex setae in parameres) and endophallites, this group is close to the *D*. *bitiensis*, *Dichotomius buqueti*, and *Dichotomius carolinus* species groups. However, the above will be evaluated in the future when the phylogenetic relationships of this diverse genus are analyzed.
